# Recent Advancements in Biomaterials for Chimeric Antigen Receptor T Cell Immunotherapy

**DOI:** 10.34133/bmr.0045

**Published:** 2024-07-15

**Authors:** Gaoyu Yu, Zhichao Ye, Yuyang Yuan, Xiaofeng Wang, Tianyu Li, Yi Wang, Yifan Wang, Jianing Yan

**Affiliations:** ^1^ School of Medicine, Zhejiang University, Hangzhou 310028, China.; ^2^ Department of General Surgery, Sir Run Run Shaw Hospital Affiliated to School of Medicine, Zhejiang University, Hangzhou 310016, China.; ^3^ National Engineering Research Center of Innovation and Application of Minimally Invasive Instruments, Sir Run Run Shaw Hospital, School of Medicine, Zhejiang University, Hangzhou 310028, China.; ^4^ Department of Translational Medicine & Clinical Research, Sir Run Run Shaw Hospital, School of Medicine, Zhejiang University, Hangzhou 310028, China.; ^5^ Department of Plastic Surgery, Sir Run Run Shaw Hospital, Zhejiang University School of Medicine, Hangzhou, 310016, Zhejiang Province, China.

## Abstract

Cellular immunotherapy is an innovative cancer treatment method that utilizes the patient’s own immune system to combat tumor cells effectively. Currently, the mainstream therapeutic approaches include chimeric antigen receptor T cell (CAR-T) therapy, T cell receptor gene-modified T cell therapy and chimeric antigen receptor natural killer-cell therapy with CAR-T therapy mostly advanced. Nonetheless, the conventional manufacturing process of this therapy has shortcomings in each step that call for improvement. Marked efforts have been invested for its enhancement while notable progresses achieved in the realm of biomaterials application. With CAR-T therapy as a prime example, the aim of this review is to comprehensively discuss the various biomaterials used in cell immunotherapy, their roles in regulating immune cells, and their potential for breakthroughs in cancer treatment from gene transduction to efficacy enhancement. This article additionally addressed widely adopted animal models for efficacy evaluating.

## Introduction

Cellular immunotherapy is an emerging cancer treatment approach, which harnesses the patient’s own immune system to target and destroy malignant cells [1,2]. Since the U.S. Food and Drug Administration approved chimeric antigen receptor T cell (CAR-T) cell therapy for B cell lymphoma in 2017, the development of cellular immunotherapy, encompassing CAR-T therapy, T cell receptor gene-modified T cell (TCR-T) therapy, and chimeric antigen receptor natural killer-cell (CAR-NK) therapy, has made significant breakthroughs and demonstrated a great potential in clinical practice [3].

CAR-T therapy involves modifying the patient’s own T cells to express specific receptors capable of recognizing and attacking malignant tumor cells [4,5]. This innovative approach has demonstrated outstanding efficacy, particularly in addressing hematological malignancies like acute lymphoblastic leukemia and non-Hodgkin lymphoma [6,7].

TCR-T therapy entails genetically altering the patient’s T cells to express T cell receptors (TCRs) that can recognize particular tumor antigens, thereby identifying and combating tumor cells [8,9]. While still in the research and clinical trial stages currently, TCR-T cell therapy has shown promise in treating some solid cancers, including melanoma and colorectal cancer [10].

CAR-NK therapy is an emerging field in cellular immunotherapy that utilizes engineered natural killer cells (NK cells) to treat cancer [11–13]. CAR-NK therapy has a number of potential benefits over CAR-T therapy. Firstly, CAR-NK cells exhibit relatively more temperate anticancer effects [14,15], for the reason that CAR-NK cells have less propensity to trigger intense, overwhelming, and excessive immunological responses, which contributes to a decrease in treatment-related adverse effects [16]. Finally, NK cells have a puissant potential of proliferation, which makes them easier to produce and manufacture in large quantities in vitro [17]. While holding promising prospects, both therapies are currently in a nascent stage of development with relatively modest scale in biomaterials researches. Therefore, this review will illustrate the biomaterials application in cellular immunotherapy with CAR-T therapy as a representative case.

With major advancements in the treatment of cancer, cellular immunotherapy is currently considered as an important therapeutic strategy [2]. However, this treatment approach still faces some challenges, such as the durability of treatment responses, side effects associated to the treatment, the longevity of therapeutic outcomes, and costs [18,19]. Furthermore, its effectiveness against solid tumors has been more challenging due to the complex tumor microenvironment (TME) and issues related to penetration and persistence of the engineered cells within solid tissues [20]. Moreover, there is a risk of on-target, off-tumor effects, where healthy cells expressing the targeted antigen are inadvertently attacked, which may lead to side effects and complications [21].

Conventional manufacture procedure of CAR-T can be elucidated as the following process: the deviation of lymphocytes through leukapheresis, target gene transduction, activation and expansion of CAR-Ts, quality control, and final administration. Dependent of in vitro storage and reaction conditions, the aforementioned steps are rather time-consuming and high-cost. Unfortunately, each procedure is confronted with unforeseeable risks and challenges. For example, a great proportion of patients with malignancy were used to receive chemotherapy, which added difficulties to collecting sufficient leukocytes for subsequent treatments. Other disadvantages incompletely include functional aberration during storage, exhaustion under in vitro activation, cytotoxicity brought by traditional transduction, and cell intolerance to TME [22–24]. Facing these shortcomings, appropriate development of biomaterials offer assistance in every procedure such as bio-instructive implantable scaffolds that allowed in vivo transduction and release of CAR-T to extensively shorten the manufacture time [25] and nanomaterials for normalizing the TME [26]. The more comprehensive application of biomaterials will be illustrated below.

Although the development of novel cellular immunotherapy strategies, such as TCR-T therapy and CAR-NK therapy, partially solves these problems, efficacy remains unsatisfactory. Biomaterials have become essential components in cellular immunotherapy, fulfilling various roles such as carriers, scaffolds, or supplementary therapeutic tools to enhance treatment effectiveness and support the functionality and longevity of engineered immune cells [27]. As effective carriers for CAR-T therapy, biomaterials are not only offering a favorable 3-dimensional (3D) milieu microenvironment for engineered T cells but also reducing nonspecific immune responses, hence ameliorating treatment-associated adverse effects and improving overall safety and efficacy [28]. Furthermore, by implementing strategic alterations, researchers have the ability to employ these substances for the purpose of transmitting immune regulatory factors or tumor-specific antigens [29]. This process ultimately enhances the precision and effectiveness of cellular immunotherapy [30].

In recent years, the study of employing biomaterials to enhance the efficacy of cell immunotherapy has advanced significantly [27,31–34]. With the purpose of objectively presenting current progresses, highlighting both innovations and shortcomings of relevant researches and serving possible future development directions of biomaterials, we cover a wide spectrum of biomaterials utilized in cell immunotherapy with primary emphasis on CAR-T and elucidate their functions in regulating immune cell proliferation and activation as well as facilitating more accurate and efficient cell immunotherapy in this comprehensive review. In the process, we focused on the role of different types of biomaterials in gene transduction, efficacy monitoring, and therapy efficacy enhancement. We also present a thorough overview of several cell carrier platforms and animal models used for evaluating the therapeutic effects in order to assist further investigations in the area of biomaterial-enhanced cell immunotherapy. Ultimately, we will provide our perspectives on the future of biomaterials in immunotherapy. In conclusion, the future of biomaterials in the realm of cell immunotherapy is exceedingly promising, and we anticipate significant breakthroughs in the treatment of cancer through the integration of biomaterials with cell immunotherapy. Moreover, with the progress of new cellular immunotherapy research, such as TCR-T cell therapy and CAR-NK cell therapy, we are also looking forward to the further integration and application of biomaterials for a wider range of cellular immunotherapy.

## Gene Transduction

Gene transduction is considered as a crucial step in the manufacture of CAR-T [35]. The effective and precise transduction of CAR gene allows engineered T cells to specifically target tumor cells while minimizing potential harm to healthy tissue [36]. A delivery system that can well condense transduction efficacy, ensure biosecurity, and maintain cost-effectiveness is urgently needed in order to ease the widespread clinical implementation [37]. As Table 1 showed, a range of strategies have been unearthed based on distinct principles and materials [38,39]. Currently, prevailing tactics utilized can be broadly categorized into 2 components: ex vivo and in vivo. In this review, we provide a concise overview of both methodologies, with a particular focus on the application of biomaterials within this field.

**Table 1.** Biomaterials application in gene transduction

Biomaterials	Type	Principals	Components	Advantages	Reference
Nanoparticles	Ex vivo	Photoporation (physical)	mRNA, bovine serum albumin, polydopamine	Biodegradable, high throughput	[74]
Bacterial magnetic particles	Ex vivo	Magnetofection	DNA, polyethyleneimine (PEI), BMP	Improvement based on PEI and wider range of cell types applied	[77]
Nanoparticles (organic-polymer)	In vivo	Receptor-mediated endocytosis	DNA, poly (β-amino ester) (PBAE) polymer, microtubule- associated sequences-nuclear localization signal (MTAS-NLS) peptide, poly (glycolic acid) (PGA)	Easy to manufacture, stable, inexpensive, and broadly applicable	[89]
Nanoparticle (Lipid)	In vivo	Receptor-mediated endocytosis	Lipid nanoparticles, mRNA	Innovate therapeutic platform for cardiac fibrosis	[95]

### Ex vivo component

A comprehensive array of ex vivo gene delivery approaches primarily emphasize the physical mechanisms. The underlying principles of ex vivo gene delivery platforms mostly involve membrane penetration, which entails the reduction of membrane perforation using external forces [40]. Various methods are applied, including electroporation [41], magnetofection [42], photoporation [43], microfluids [44,45], microinjection [46], and sonoporation [47].

**Fig. 1.**
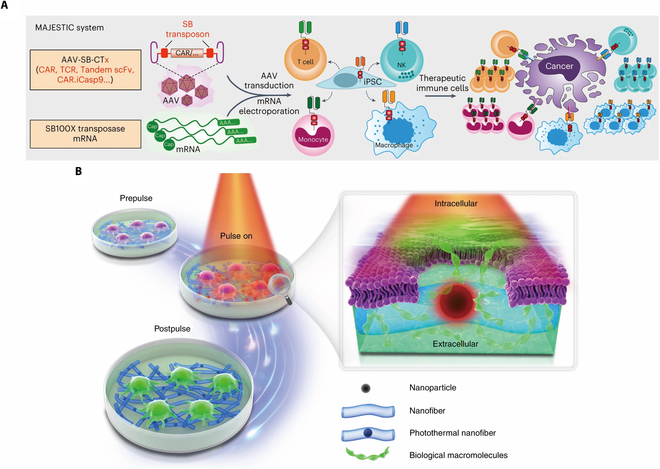
(A) Schematics of the hybrid AAV-Sleeping Beauty (AAV-SB) construct, SB100X mRNA electroporation and CAR T/NK/macrophage/iPSC generation, which formed 2 core components of mRNA AAV–SB joint engineering of stable therapeutic immune cells (MAJESTIC) system. Copyright © 2023, Lupeng Ye et al., under exclusive license to Springer Nature Limited [55]. (B) A schematic overview of intracellular delivery by membrane permeabilization with photothermal nanofibers. Copyright © 2021, Ranhua Xiong et al., under exclusive license to Springer Nature Limited [69].

Electroporation serves as a fundamental technique for transducing CAR gene by physical means, playing a pivotal role in diverse delivery platforms [48]. In the configuration established by bulk electroporation, the conducting buffer solution contains the suspended cell and gene carrier between 2 connecting electrodes. The cell membrane, being an electrical insulator with stable transmembrane potential, is capable of being influenced by an external electric field [49]. This external electric field can cause an increase in the potential difference across the membrane, reaching a threshold that leads to breakdown of membrane [48]. Consequently, temporary pores on membrane are formed enabling the diffusion of gene vectors [50]. Electroporation is a highly efficient method with clinical transformation value, capable of achieving high throughput, regardless of the constraints imposed by cell type, cell cycle, or biological characteristics of carriers [51]. The transposon system in mRNA have been extensively investigated as approaches for gene transduction in conjunction with electroporation [52–54]. As for transposon systems, the Sleeping Beauty and PiggyBac are relatively stably established [53]. Figure 1A showed schematics of the hybrid adeno-associated vector (AAV)–SB construct, SB100X mRNA electroporation, and CAR T/NK/macrophage/induced pluripotent stem cell (iPSC) generation [55]. Combined with gene-transduction techniques, human iPSC is capable of converting into patient-specific progenitor or functional cells even tissue and organs, among which CAR-T involved. The whole procedure for iPSC-related CAR-T study is composed of iPSC preparation, CAR transfection, and differentiation [56]. Initially, somatic cells principally extracted from peripheral blood are subject to reprogramming triggered by virus-mediated delivery or nonintegrating gene transfection and then reverse into iPSCs [57,58]. Subsequently, CAR gene is introduced into iPSCs through various pathways including lentivirus [59,60], electroporation, with zinc-finger nuclease editing [61] or PiggyBac transposon systems [62]. CAR-bearing iPSCs are then cultivated within a specially designed culture medium and exposed to selected growth factor and cytokines that guide their predetermined differentiation pathways following strict timing. To date, researchers have provided detailed protocols for iPSC-NK [63], iPSC-T [59], iPSC-macrophages [60], and iPSC-monocytes [64]. These systems exhibit enhanced cargo capacity and biosecurity as they are proficient in mediating steady integration of DNA into the genome of the host. In the meanwhile, it is worth noting that these transposon systems maintained as plasmid DNA demonstrates cost-effectiveness in terms of manufacturing and holds promise for potential scale-up production [65]. The RNA electroporation enables the production of the CAR construct for a period of 7-d cycle, thereby establishing a basis for further extensive examination of single-chain variable fragments and the safety of CAR constructs [66,67]. Nevertheless, electroporation has faced significant criticism due to its possible cytotoxicity, including membrane damage, lipid peroxidation, and high-rate cell death [68]. These effects are believed to be caused by inappropriate physical parameters used during the process.

Fig. 1B demonstrated a schematic overview of intracellular delivery by membrane permeabilization with photothermal nanofibers [69]. Photoporation could serve as an alternative, operating on the similar concept of membrane disruption [70]. Photothermal sensitizers, such as gold nanoparticles (AuNPs), have the ability to transform light energy into thermal energy, causing the reduction of water evaporation in the vicinity and the creation of vapor nanobubbles [71]. The vapor nanobubbles rapidly undergo collapse, resulting in the generation of shockwaves that induce the formation of reversible pores in the membrane [70,72,73]. To address the issue of nonbiodegradability of AuNPs, a team led by Braeckmans developed biocompatible polymeric nanoparticles as a potential solution. They synthesized the polydopamine nanoparticles as a substitute for AuNPs and coated the surface of polydopamine nanoparticles with bovine serum albumin to booster the colloidal stability and cell affinity. Following laser irradiation, the effective transduction of mRNA expressing FD500 and enhanced green fluorescent protein was observed in human T cells, HeLa cells, and Jurkat cells [74]. It is worth mentioning that the collaborative integration of electroporation and microfluidic systems can contribute to the generation of higher localized and concentrated electrical fields. It becomes feasible by the utilization of controllable microfluidic channels, which in turn promote cell viability [75].

Other methods may potentially be employed for CAR-T gene transduction. Magnetofection is a promising approach for application in CAR-T therapy, with a close association to biomaterials. It enables the complex composed of nucleic acids and magnetic nanoparticles to be absorbed by endocytosis or pinocytosis under the guidance of external magnetic field [76]. Recently, bacterial magnetic particles (BMPs) have been displayed for future application. Fang et al. conducted research showing that the BMPs-PEI gene carrier exhibited higher transfection efficiency in Cos-7, HeLa, and HepG2 cell lines. This vector system is biocompatible with low cytotoxicity when delivering DNA into primary cells [77].

### In vivo components

The conventional ex vivo strategy involves the extraction of T cells from the blood of patients, followed by a series of steps including activation, transduction, expansion, and ultimately autologous reinfusion. The production of these products is characterized by a high degree of customization, necessitating labor-intensive, time-consuming, and expensive industrial engineering processes. The technology is also facing criticism due to certain flaws. One such flaw is the potential transfer of CAR into cancer cells, which can occur when these cells infiltrate the bloodstream and evade detection by T cells.

The utilization of in vivo methods for gene transduction has developed for therapeutic purposes. Complex procedures have been circumvented in favor of an exclusive reliance on particle infusion. To a significant extent, the occurrence of side effects, such as graft–host reaction and reduced viability of CAR-Ts, was effectively mitigated. Vectors that are commonly employed encompass both viral and nonviral types. The distinctions between nonvirus and virus are enumerated in Fig. 1A and B. Both of these approaches have a shared basic principle, which is to provide the carriers with the ability to selectivity target certain subsets of immune cells. The identification of specific cell markers is a commonly employed approach, with the use of specialized materials or structures such as single-chain variable fragments or designed ankyrin repeat proteins enhance stronger affinity to particular subsets.

The viral-vector field is greatly concerned with retrovirus families, such as lentiviral vectors (LVs), γ-retrovirus, and AAVs. Retroviruses has the capacity to do reverse transcription, wherein the viral RNA genome is converted into complementary DNA (cDNA). This cDNA is then integrated into the chromosomal DNA of the host organism [67].

LVs are widely employed in clinical research, constituting approximately 54% of the methods to induce T cell activation. The determination of transduction preference, also known as tropism, is governed by the viral envelope protein, which is intricately associated with the process of pseudo typing. As for LVs, the replacement of the surface protein with glycoprotein G derived from vesicular stomatitis virus enables the internalization of LVs by various categories of cells through the mediation of low-density lipoprotein receptor. Conversely, γ-retroviral vectors have the ability to mimic the amphitropic murine leukemia virus as well as comparable approaches [67]. Both γ-retrovirus and lentivirus have been found to contribute to increased expression and improved transduction efficacy [78].

Additional groups of viruses were also documented. AAVs, which are nonenveloped viruses containing single-stranded DNA, enter cells through a receptor-mediated mechanism. Through endosomal escape, synthesis of second-strand DNA (ds DNA) also enters the nucleus by a process of endosomal escape. Once inside, it undergoes the synthesis of ds DNA. Subsequently, the ds DNA has the potential to either integrate into the genome or persist as an epistome [79]. Compared with LVs, the transduction span of the vector in activated lymphocytes was notably shorter. Capsid engineering is a frequently employed method of modification. In a recent study, researchers successfully achieved efficient transduction in preclinical models of Trac-targeted CAR-Ts by employing a developing AAV variation [80].

Although virus-based vectors exhibit remarkable transduction efficiency, it is still hindered by certain shortcomings. The immunological response of the host was still likely to be triggered by all viral proteins. Due to gene integration behavior, clinical monitoring process should place a premium on risks related to intersectional oncogenesis. Different types of viruses exhibit distinct preferences for insertion, as seen in patients who were exposed to lentivirus-mediated transduction. In these cases, the lentivirus tended to insert itself into the introns of genes that were actively involved in transcription [67]. Consequently, these patients experienced reduced T cells expansion and insufficient antitumor efficacy [81]. Simultaneously, it should be noted that the aforementioned viral vectors exhibited a constraint in terms of payload capacity, in particular limited to 8 kb which poses a challenge for supplementary genetic manipulations.

### Nonvirus delivery platforms

Nonvirus delivery platforms rely extensively on biomaterials, including lipid, polymers, and inorganic nanoparticles, which are customized based on the specific properties of the materials and practical requirements. Herein, the nonvirus delivery platforms are classified into 2 categories, including organic and inorganic, in order to provide a more comprehensive understanding.

#### Polymers

**Fig. 2.**
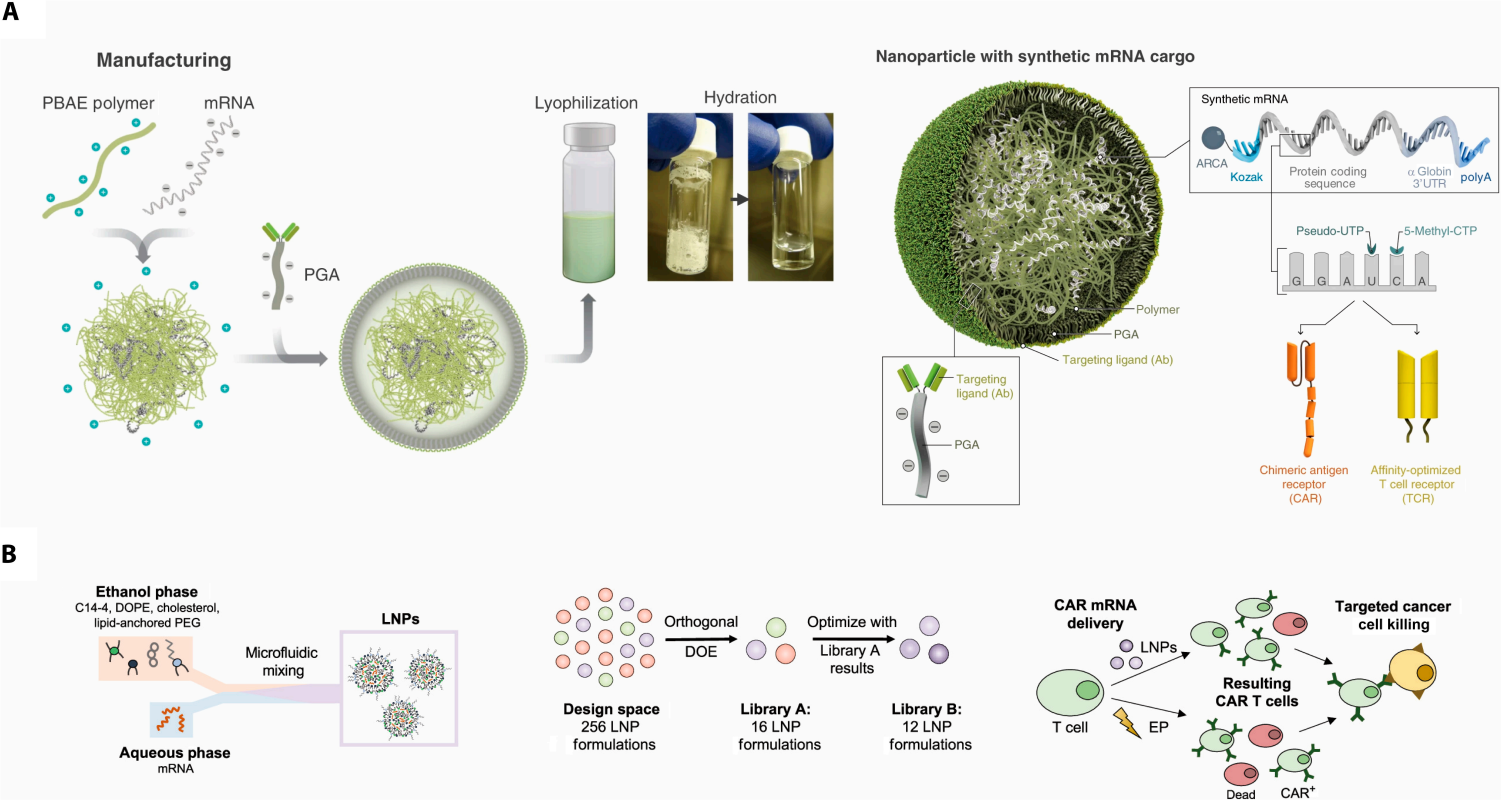
(A) Schematic of the T cell-targeted in vitro transcribed mRNA nanocarrier. Copyright © 2020, N. N. Parayath et al. [90]. (B) Schematic of LNP synthesis including the components used to make LNPs via microfluidic mixing and the expected resulting structure. Copyright © 2022, American Chemical Society [94].

The development of polymer-based carriers involves the utilization of both electrostatic and covalent interactions [82]. Extensive research has been conducted on polycations. These synthesize of these polycations is principally achieved using polyethylenimine (bPEI) [83], poly(2-dimethyl) aminoethyl methacrylate) (pDMAEMA) [84], polyamidoamine (PAMAM) [85,86], and poly (β-amino ester) (PBAE) [87]. The polycation can be synthesized into different geometric configurations, such as linear-branched (comb), cyclic-branched (sunflower), or star-shaped. According to the findings, variations in transduction effectiveness and cell viability can be attributed to differences in core size, branch length, and numbers. Nonetheless, it is important to note that a majority of polycations still fail to achieve a satisfactory transfection efficiency [88]. Furthermore, there exists an extra mentality of designing based on polymer. Stephan et al. made significant contribution to this field. They developed an innovative nanoparticle in order to achieve the in situ reprogramming of T cells. The anti-CD3e f(ab’)2 fragments were conjugated to the surface of PBAE nanoparticles to specifically target to the T cells. The plasmid DNA encoding 194-1BBz CAR, which were aimed at leukemia cancer cells, and iPB7 transposase derived from PiggyBac transposase system were encapsulated altogether into nanoparticles. To reduce the occurrence of nontarget combinations, a negatively charged polyglutamic acid was introduced as a coating agent for nucleic acids. The utilization of bioluminescence imaging facilitated the observation that the CAR gene was effectively transferred to CD3 T cells precisely, as evidenced by the detection of coexpress click beetle red luciferase. In addition, the longevity of mice and the growth of tumors demonstrated a comparable antitumor capability to that of traditional ex vivo engineered CAR-Ts with minimal toxicity [89]. In 2020, this team successfully achieved nanoparticles as a means to deliver mRNA. Similar to previous structure, the nanoparticle is comprised of PBAE that encapsulates mRNA, while concurrently including a target ligand CD8 in conjunction with poly (glycolic acid) (PGA), as shown in Fig. 2A [90]. A series of findings demonstrated successful delivery of mRNA by the nanoparticle, resulting in sustained CAR expression for 1 week following each injection. The survival duration of mice with prostate tumors was prolonged for 40 d, and effective infiltration of CAR-T into solid tumor was found by fluorescence imaging. It is hypothesized that the persistence of tumors can be attributed to their down-regulation of target antigens. The nanoparticle simultaneously displayed the ability of intracellular HBV core antigen recognition while delivering TCR transgene. In contract, most in vitro transcribed mRNA-encoding CAR were only capable of recognizing antigens produced on the cell surface.

#### Lipid

There are further studies that investigate the potential utilization of lipid nanoparticles. The production of polymer nanoparticles often exploits the utilization of electrostatic connections between lipid molecules with positive charges and nucleic acids with negative charges. This interaction leads to form a complex, which is later internalized by cells and releases its cargo. It has been found that reengineered T cell immunotherapy holds promise for the reversal of cardiac fibrosis [91]. Figure 2B demonstrated an orthogonal design of experiments for optimization of lipid nanoparticles for mRNA engineering of CAR-Ts. Lipid nanoparticles (LNPs) consist of 4 main components: ionizable lipid, cholesterol, phospholipid, and PEGylated lipids [92]. Genetic materials including small interfering RNA (siRNA), mRNA, and plasmid DNA [93] are encapsulated into the mixture of components mentioned above through methods like microfluid mixing [94]. Take the LNP siRNA system as an example; after venous administration, it will combine with ApoE and then enter the hepatocytes through endocytosis mediated by ApoE-related ligands17 and 19. Under the pH environment of endosomal, large proportion of ionizable lipid will be protonated and attracts the endogenous anionic lipid which leads to the formation of endosome-disrupting nonbilayer structures and release of contents. It is worthwhile to mention that the LNP can be targeted toward various specific cells or tissues as long as equipped with therapeutic proteins including monoclonal antibodies. Epstein. et al. created an experimental immune therapy utilizing in vivo transduction for the treatment of cardiac fibrosis [94]. The LNP was coupled with a protein that selectively binds to CD5, a molecule that plays a vital role in cellular processes. This protein is loaded with mRNA encoding CAR gene, which specifically targets fibroblast activation protein and Cre recombinase. Bioluminescence imaging indicated that only the experimental group, which received the injection of CD5/LNP-FAPCAR, exhibited detectable and consistent CAR-T subsets. The echocardiography surprisingly showed a notable improvement in cardiac function, namely in the left ventricle diastolic function, following the administration of temporary CAR-Ts [95]. However, due to the disability of genomic integration, mRNA was restricted to cytoplasm and prone to be diluted or be decomposed, triggered only transient expression of fibroblast activation protein.

By considering the existing methods employed for in vivo gene delivery, it is foreseeable that the efficient generation of targeted CAR-Ts will eventually be achieved. There remain some unresolved issues pertaining to in vivo transduction that warrant significant attention for future improvement. The efficacy of in vivo transduction is currently impeded by off-target effects, which occur when subsets of cells expressing the same markers are mistakenly targeted, resulting in an insufficient number of effective CAR gene deliveries and an increased risk of unexpected immune functional interference. In contrast to the conventional ex vivo transduction approach, the utilization of in vivo transduction results in a significantly lower production CAR-Ts by many orders of CAR-Ts by many orders of magnitude. This reduction can be attributed to factors such as gene dilution, degradation, or inactivation. Immune reaction to the carrier or its binding protein, as well as the process of phagocytosis, has a significance in reducing carrier efficacy. In clinical practice, it is recommended to administer subsequent injections, as this helps to sustain an optimal concentration level and reduce the likelihood of tumor reoccurrence. Despite that, it is likely that insertional mutagenesis occurs in the most significant integration systems. We have yet to make significant progress.

#### Other materials

Actually, in addition to common vector based on lipid or cationic polymers, other attempts have been made to explore more potential transfection tools. The cell penetrating peptides (CPPs) is the first to mention. Researchers found that CPP with amphipathic RALA motif repeat could deliver mRNA into dendritic cells (DCs) [96]. However, the mechanism underlying remains unknown and is considered to relate to micropinocytosis or lipid bilayer disruption [97]. Besides, nanostructure, which encompasses nanostraws, nanowires, and so on, is representative of one alternative to directly pierce the cell membrane and transfer nuclei acid. It embraces various cargos including siRNA, pDNA, and other molecules [98]. Shokouhi et al. developed a nonviral electroactive nanoinjection platform. Configured under low voltage, the electroactive nanotubes enabled surprising delivery efficacy and expression with cell viability maintaining high-level [99]. Such combination gives a possible answer to overcome the disadvantages of electroporation and nanostructure. It is of note that both methods mentioned above still lack a clear mechanism and suffer from the disadvantage of low transfection efficacy. Other research reported recombinant PP7 virus-like particles, a vector offering more stable properties and protecting the mRNA from degradation compared to CPPs [100].

## Monitoring the Efficacy of CAR-T

The conventional monitoring of effectiveness of CAR-T therapy typically focuses on the decrease in tumor size or alleviation of clinical symptom in patients. However, this approach often fails to promptly detect the off-tumor response or any adverse events associated with the therapy. Hence, as Table 2 shows, there is a pressing need in clinical practice for the observation of T cell growth, activation, and routine trafficking in order to assess the early stage of response and provide timely intervention [101,102].

**Table 2.** Biomaterials application in CAR-T imaging

Biomaterials	Imaging method	Components	Model	Results	Possible application	Reference
Nanoparticle	MRI	Ultrasmall superparamagnetic particles of iron oxide (USPIO); coating: low-molecular- weight amino alcohol derivative of glucose	GBM (glioblastoma)	Increasing hypointensity signals detected in susceptibility-weighted imaging MRI after 3–14 d following injection on model	Early therapeutic effects validation and T cell tracking	[104]
Nanoparticle	NIRF, PET	Silica, 89Zr (protamine, heparin)	SKOV3: hCEA(+)	Nongenomic cell trafficking up to 1 wk	Direct cell labeling and nanodrug delivery	[105]
Nanoparticle	Fluorescent imaging, CT	G5.NH2, AuNPs, Fluo-4	HLA-A2+ SKMEL-23	CT imaging revealed precise distribution and localization of CAR-T with increased fluorescence signal within 24 h	Dual-modal monitoring of activation and distribution	[106]

In the early stages of magnetic resonance imaging (MRI) tracking of immune cells, gadolinium chelated with paramagnetic contrast agents, which provide positive contrast in T1-weighted sequences. The development of gadolinium was constrained due to its probable toxicity. Subsequently, paramagnetic chemical exchange saturation transfer was introduced. Nevertheless, it was shown to possess inadequate sensitivity. In the early 1990s, superparamagnetic iron oxide (SPIO) finally arose, originated from the iron oxide that was first used for ex vivo cell tracking. SPIO can strengthen the negative contrast in T2-weighted sequence of MRI, whose radius is adjustable from nanometer to micrometer scale and with improved sensitivity. Different transfection methods were created as a result of variable labeling efficiency of SPIO, including the combination with monoclonal antibody and the mixture of ferumoxytol, protamine, and heparin. Besides, it has been reported that other transfection methods such as electroporation, various transfection agents (e.g., poly-L-lysine, lipofectamine, and protamine sulfate), and conjugation of HIV-1 transactivator peptides may be toxic to cells, which could reduce the viability, phenotype, and efficacy of transfected cells [102,103]. Combined with biomaterials, researchers offered a different vision of better transfection. Liu et al. synthesized a novel superparamagnetic nano-sized iron-oxide particle, IOPC-NH2 series, to increase safety. The particles are encapsulated in polyethylene glycol (PEG), which lacks of immunogenicity and antigenicity. Without the use of the aforementioned transfection agents, the particle demonstrated a transfection efficiency of over 90%. No discernible difference was found in the expression of CD62L and CD25 between IOPC-NH2 labeled and unlabeled normal rat T cells in the rat model. Additionally, the immune response toward LPS stimulation was identical in IOPC-NH2 labeled Jurkat cells and unlabeled Jurkat cells, indicating the hypotonicity of the particle [104].

Based on the prior research on the incorporation of endothelial progenitor cells labeled with ultrasmall superparamagnetic particles of iron oxide (USPIOs), Zhang et al. developed the amino alcohol derivatives of glucose-coated nanoparticles. These nanoparticles were utilized to track the infiltration and duration of CAR-Ts in a manner with noninvasive procedures. The optimized concentration of iron, specifically 37.5 μg/ml, was selected based on the comparatively high viability of CAR-Ts. The transmission electron microscopy analysis indicated the presence of black particles in the cytoplasm of CAR-Ts that were labeled with USPIO, in contrast to the nonlabeled groups. This observation suggests that the complexes exhibit a high level of effectiveness in terms of endocytosis [103]. Furthermore, the application of subsequent CD11b and Iba-1 immunostaining effectively excluded the possibility of nonspecific endocytosis. MRI was employed to monitor the growth of glioblastoma multiforme (GBM) xenografts, Susceptibility-weighted imaging (SWI) was performed on days 3, 7, and 14, revealing the progressive emergence of hypointense signal following the injection of a minimum of 1 × 10^5^ labeled CAR-Ts. Nonlabeled cells showed no hypointense signal. The diffusion-weighted imaging MRI imaging revealed a noteworthy drop in the Ktrans value and a substantial increase in apparent diffusion coefficient in USPIO-labeled CAR-Ts, as compared to the control group, when administered after 3 d. This finding suggested that the early stages of vascular and cellular changes can be detected using these imaging modalities [104]. This discovery was further substantiated by subsequent histological sections and SWI imaging. Additional contrast agents (e.g., fluorine-19 perfluorocarbon) have been reported with a promising potential for future applications. This is due to their low toxicity and negligible interference with background signals. The comparison between MRI and positron emission tomography (PET) is presented in Table 2.

Nuclear medicine imaging has also been extensively explored, with the direct and indirect labeling systems emerging as the 2 primary methods. Prior to the emergence of PET imaging, significant efforts were focused on the application of single-photon emission computed tomography for T cell direct-labeling tracking. The radionuclides 111In and 99mTc were frequently used for ex vivo cell tracking but without enough labeling stability. 111In-oxine and 99mTc-HMPAO solved the problem relatively by promoting the diffusion into cells and combination of isotopes and protein in cytoplasm. However, the requirement for a significant increase in dosage due to the sensitivity and loss through efflux of radioisotopes demanded huge dose of addition, which reduced to the cytotoxicity. The presence of photon attenuation and scattering posed a significant challenge in accurately quantifying the signal intensity. The all above the defects hindered the pace of further advancements in single-photon emission computed tomography. In contrast, PET has emerged as a viable alternative due to its superior tumor background ratio. The underlying principle of PET involves the emission of radionuclides, which subsequently produce positrons and neutrinos in order to maintain stability. Following the annihilation of a positron with an electron, the resulting photons are emitted and subsequently detected by specialists. To address the issue of the relatively short half-life of radionuclides, researchers have explored the use of chelating agents, such as methyl 4-methylbenzene-1-sulfonate (PTSM), to slow down the rate of decay. However, this approach still faces challenges because of the significant efflux of isotope.

**Fig. 3.**
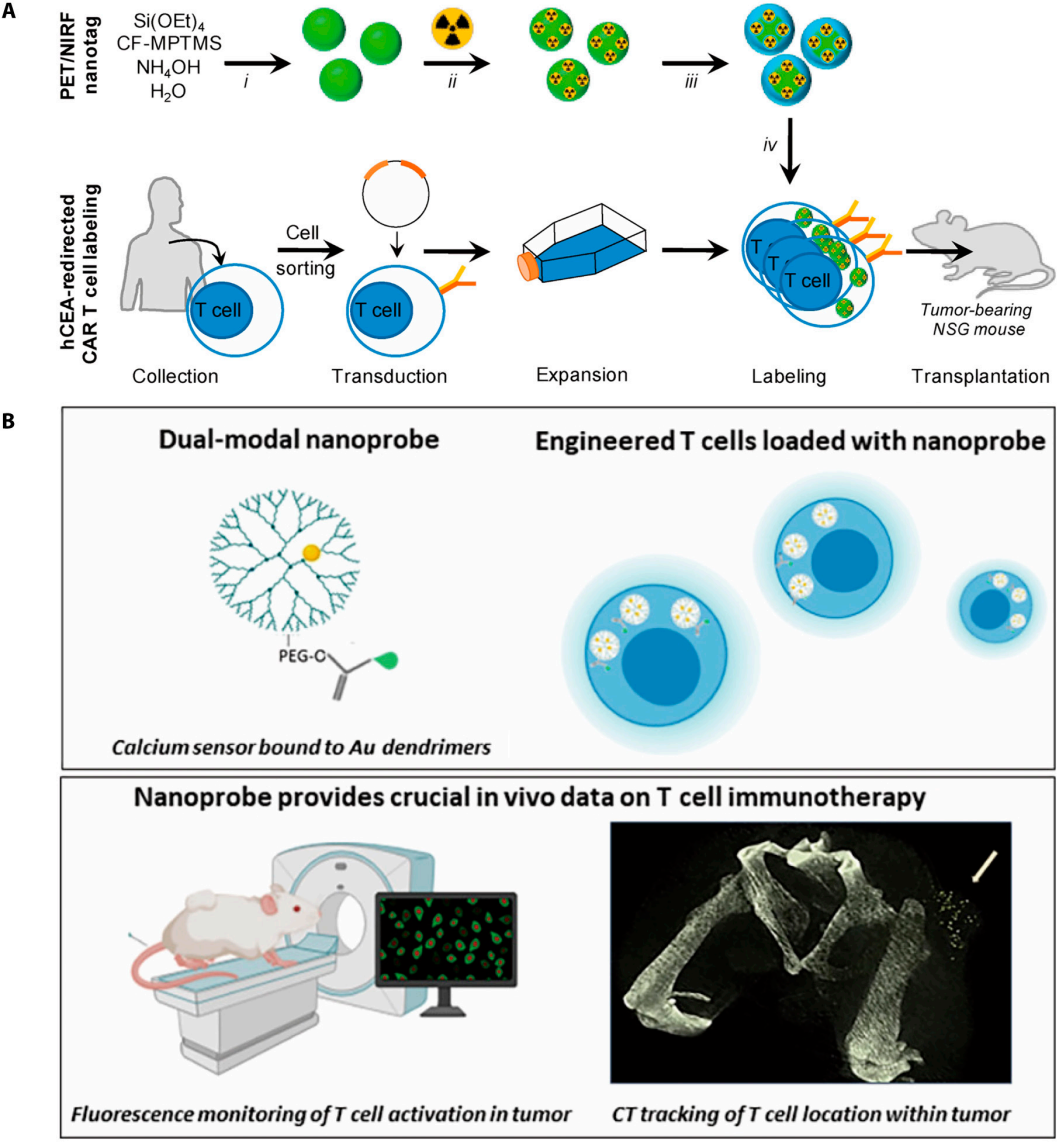
(A) Schematic of labeling strategy to monitor T cell distribution following transplantation in tumor-bearing mice. Copyright © 2020 Elsevier Ltd [105]. (B) Schematic of the dual-modal nanoprobe loaded engineered T cells, which enables fluorescence and CT imaging for real-time monitoring of the cells in vivo. Copyright © 2020 Elsevier Ltd [106].

Harmsen et al. designed a novel nongenomic imaging technique utilizing dual-modal PET/near-infrared fluorescent (NIRF) nanoparticles to obtain a sustaining observation in vivo for 1 week following immune cell infusion, as shown in Fig. 3A. By incubation with the NIRF silica nanoparticle labeled with 89Zr as well as protamine and heparin, the CAR-Ts were administrated peritoneally and intravenously. PET showed the concentrated signal in the lungs and liver following intravenous delivery, and in the liver and spleen following peritoneal administration. The imaging results obtained on day 14 using NIRF demonstrated a strong correlation with PET. Furthermore, the technique employed to mark the CAR-Ts demonstrated significant clinical translation potential, as a majority of these methods have been validated for clinical application and are already in widespread usage. Nevertheless, the nanoparticle continued to experience apoptosis following a 1-week infusion. However, it has been observed by researchers that the preceding analysis of the peritoneal tumor section has demonstrated that the nanoparticle released was effectively internalized by tumor cells, indicating that this system has the potential to be applied to better nanodrug delivery [105]. The process of indirect labeling necessitates modifications to genetic materials, which facilitates the production of certain reporter proteins. The reporter gene provides enhanced stability in expression and extended duration of monitoring. The hindrance of transduction-related risks on cell functionality and immunogenicity, along with the ethical and moral concerns, has unfortunately impeded its advancement.

In vivo optical imaging encompasses several techniques, including as fluorescence imaging, bioluminescence, confocal and 2-photon microscopy imaging, as well as diffuse optical tomography. The categorization system can also be broadly classified into direct and indirect. The direct labeling system requires the combination between dye and cell, such as the near-infrared (NIR) dye DiD system used for labeling NK-92 cells and the avidin–biotin system. The indirect system necessitates the alteration of the genome. However, the research on the application of biomaterials in optical imaging is limited. Furthermore, additional attempts of monitoring CAR-T have been undertaken. Popovtzer et al. developed an innovative and versatile nanoprobe by using PAMAM dendrimers as carriers, integrating AuNPs with a fluorescent calcium sensor, and forming a dual-modal nanoprobe. The hyperbranched nanopolymers, known as 5 PAMAM, exhibited a modifiable structure based on the applied load. AuNPs have been widely regarded as a desirable contrast agent for computed tomography (CT) due to excellent x-ray attenuation, and many studies revealed the correlation between the activation of T cells and intracellular calcium ion concentration increase. The examination conducted by researchers using enzyme-linked immunosorbent assay revealed that the labeled CAR-Ts exhibited comparable tumor necrosis factor-α secretion ability and immunogenicity in comparison with control groups. The results of in vivo fluorescent imaging indicated that the fluorescent signal peaked at 4 h after administration and remained detectable for nearly 24 h. This temporal profile of fluorescence provides valuable insights into the activation of T cells. As shown in Fig. 3B, the CT scan conducted 24 h after administration demonstrated that T cells labeled with nanoprobe showed a remarkable stronger signal compared to nonlabeled cells. This signal strength effectively displayed the distribution of engineered cells [106].

In overall, the monitoring methodologies of tracking CAR-Ts demonstrate substantial potential. However, there remain certain issues that require attention, for example, the signal attenuation caused by cell lysis or exocytosis, which hinder the feasibility of prolonged observation. There is still a significant distance to be covered in order to achieve a stage where a method can be considered safe, accurate, real-time, and cost-effective.

## Efficacy Enhancement

In recent years, biomaterials have been increasingly used as effective carriers in cellular immunotherapy for CAR-T therapy and yielded good results, as shown in Table 3.

**Table 3.** Partial biomaterials for increasing efficacy

Type	Materials	Adjuvant/combined therapy	Model	Efficacy	References
Nanoparticle	Protein nanogels	IL-15	B16F10	Controlled release and higher doses without toxicity	[125]
	Lipid gel	aPD-L1, IR820	4T1, B16F10	NIR-mediated controlled release and enhanced recruitment and cell viability	[120]
	Dibenzocyclooctyl (DBCO) and N3-group (through bio-orthogonal reaction)	Mild photothermal therapy	Raji	Fluorescent tracing and TME remodeling	[111]
	Poly (lactic-co-glycolic acid)	Anti-PD-L1 antibody, R848	SK-OV3, B16F10	Increased cytotoxicity and activation	[108]
	HA@Cu2-xS-PEG	-	NSCLC	TME remodeling	[162]
	Immunomagnetic beads	-	CD19-SPCA1	Precision targeting and better penetration ability	[159]
Hydrogel	Alginate	Metformin	HGC-27	Metabolic regulation and TME remodeling	[141]
	HA	IL-15, platelets conjugated with PD-L1	WM115	Suppression of recurrence and distant growth of tumor	[138]
	Gelatin, methacryloyl groups	IL-2, IL-7, IL-15	Jurkat, HEK-293T, B16F10	Photocurable-controlled release of CAR-T	[31]
Microneedle	4-Arm-PLGA-Acry, AIBN, PLGA, TEGDA	-	WM115	Local delivery	[151]
Nickel titanium thin films	Nitinol	-	OVCAR-3	Prevent tumor ingrowth and occlusion	[153]

### Nanoparticles and microparticles systems

Nanoparticles and microparticles, which are extensively studied biomaterials, play a crucial role in enhancing the effectiveness of tumor eradication. The particles possess the capacity for adjustable size and deformability, which may be customized to meet various tissue-infiltration needs and unique physical and chemical properties. This characteristic serves as a basis for materials development, as it is derived from a wide range of sources. The charge effect is a significant factor that impacts the absorption by antigen presentation cells, subsequently resulting in the activation of CAR-Ts. In addition, surface functionalization is an effective technique that may be utilized to achieve targeted cell binding. To provide a concise overview, extant studies pertaining to this subject matter can categorized into organic-based and inorganic-based. It is important to note that in practical applications, different materials often combine with one another to create a convergence of distinct advantages. The effect is of major concern because it influences antigen-presenting cell uptake, which leads to CAR-T activation downstream. Furthermore, surface functionalization, as a powerful tool, can fulfil the targeting of certain groups of cells while also serving as a crucial design thrust. To provide a concise overview, existing researches relevant are classified into organic and inorganic categories. However, it should be noted that in practice, multiple materials actually combine to create unique advantages [103].

### Organic materials

The polymers-based biomaterials dominate in terms of numbers developed. Diversified materials provide more complex structures, surface functional units, and special qualities to produce desired results, such as controlled drug release and degradation in response to a specified environmental pH value. Conversion under TME circumstances, extended retention, and reduced biotoxicity could be feasible design foothold in the quest to enhance therapeutic efficacy. All of these traits contribute to transformation of the entire CAR-T therapy process to our advantage, from enhanced cell expansion and activation to a change in the harsh TME, and make polymer-based biomaterials increasingly appealing.

Kim together with his team collaborated to develop a working integrated nanosystem. The nanosystem contained nanoparticles loaded with doxorubicin, as well as biodegradable photobleaching-resistant fluorescent polymer (BPLP)-polylactide copolymers (BPLP-PLAs). These copolymers were equipped with pH-sensitive linkers, allowing them to attach to the surface of T cells through click chemistry. Upon exposure to the mild acid TME, the linkers underwent cleavage, resulting in the dissociation of nanoparticles loaded with doxorubicin from CAR-Ts. Subsequently, these nanoparticles initiated a controlled release of medicines, therefore augmenting the cytotoxic effect. The subsequent investigations provided justification for the significant protection of normal cells by specific attachment of nanoparticles to CAR-Ts using bio-orthogonal click reaction. The inclusion of BPLP-PLAs in the system resulted in the simultaneous acquisition of an additional modality of imaging [107]. Other studies have also observed a comparable structure with a distinctive mechanism for enhancing the antitumor efficacy. Duwa et al. pioneered the development of nanoBiTEs. It involved poly (lactic-co-glycolic acid) nanoparticles loaded with R848 and afterwards coupled with anti-CD3 and anti-programmed cell death ligand 1 (PD-L1) antibodies. Extensive research has been conducted on poly (lactic-co-glycolic acid) nanoparticles, which have been demonstrated to possess biodegradability, biocompatibility, and the ability to undergo easy surface modification. These bispecific nanoparticles effectively achieved local immune-stimulation using a reinforcement strategy which referred to blocking of immune check point and promoting the DCs maturation [108].

The inherent variability and modifiability of polymer structures enable the effective integration of many therapeutic components, resulting in an outcome that exceeds the cumulative effects of each individual constituent. Phototherapy encompasses 2 modalities, namely photothermal therapy and photodynamic therapy. The photothermal therapy method causes cell death by increasing the temperature of the immediate surroundings, taking advantage of the vulnerability of cells to heat. On the other hand, the photodynamic therapy technique depends on the generation of the reactive oxygen species to induce cell death [109]. The effectiveness of phototherapy has been constrained due to the absence of safer and more efficient photothermal agents with higher tumor cell selectivity. Polymeric nanoparticles have been well recognized as promising photothermal agents due to their high light absorption, effective photothermal conversion, and accurate selectivity [110]. The team led by Cai reported a study on indocyanine green nanoparticles engineered CAR-T biohybrids. Indocyanine green is a classical NIR absorbing sensitizer with excellent photothermal conversion efficiency [111]. Based on the findings from immunofluorescence imaging and other experimental outcomes, it was determined that integration of mild photothermal intervention with indocyanine green nanoparticles effectively led to the remodeling of the TME and facilitated the recruitment and infiltration of CAR-Ts. Notably, the enhanced clearance of tumors was observed [112].

The utilization of polymer-based nanoparticles is a desirable approach for tailoring therapeutic interventions in response to the complex and individualized conditions of the innate environment in patients. The emerging field of biomimetic nanotechnology and glycoengineering represents a significant advancement in engineering capabilities. However, it is advised that the biosafety and biodegradation of organic reagents and industrial production should be thoroughly assessed to prevent the potential accumulation of cytotoxicity [103].

With the exception of magnetic materials, lipids dominate in nanocarriers development. Considered as nontoxic and biodegradable with low immunogenicity, the materials have several advantageous properties, including the ability to undergo extensive surface modification, size regulation, and adaptive encapsulation. These differentiating traits include both hydrophobicity and hydrophilicity, which are beneficial for accommodating different types of payloads. It also possesses a low production cost and utilizes scalable production technique [113,114]. The administration of immunoregulation medications to specific tumor sites is a well-known approach in reversing the TME. Among the several ways employed, lipid-nanocarriers have emerged as the preferred alternative due to their desirable properties. One exemplary instance is proposed by a research team led by Irvines. They utilized the immunoliposomes to deliver the small molecule, namely the inhibitor of transforming growth factor-β. Transforming growth factor-β is known as an immunosuppressive cytokine and is related with the activation of cancer-associated fibroblasts [115]. The purpose of utilizing immunoliposomes was to modify the TME. PEG was used as a coating agent for the liposome to hinder any potential interaction between the lipid layer and plasma proteins [116]. Conventional lipid materials administered intravenously are criticized for short residence period in circulation because of macrophage phagocytosis [117]. PEG molecules could provide liposomes with protective hydrophilic layer thus reducing the aggregation and interaction with blood components, which increases the period in circulation. However, pegylation also bring about unsatisfactory influences, including undermining the efficacy of targeted cell recognition and endosome escape [118]. Additionally, previous experiments reported that lipid-based nanoparticles was associated with the activation of complement system through alternative and classic pathways and finally resulted in pseudoallergy, infusion reaction, and acute hypersensitivity [119]. There is also growing concern about the reproducible fabrication formulation of lipid nanoparticles since minor deviation could lead to drastic change of properties in terms of large-scale standardized production [116]. The similar structure is seen in Stephen’s study as well. They successfully synthesized nanoliposomes that were coated with tumor-targeting peptide iRGD on their surface. These nanoliposomes were designed to encapsulate a phosphoinositide 3 kinase inhibitor, which acts as an immunosuppression, and a-GalCer agonist, which functions as an immunostimulant. The results showed enhanced homing efficacy, prosperous cell proliferation, and reduction in tumor size [120].

Organic biomaterials derived from lipids can manifest in various forms, outside the scope of nanoparticles. Considering the injectable lipid gel (LG) as an illustrative example, Luo et al. developed an integrated drug delivery system, known as the mild photothermal-sensitized immunotherapy system, in which an injectable LG plays a crucial role. The LG is composed of a combination of soybean phosphatidylcholine and glycerol dioleate, which have been deliberately manipulated to induce a reversible morphological alteration. By incorporating the photothermal agent IR820 and the anti-PD-L1 antibody, the LG displayed the capability of photothermal sensitivity and immunoregulation [121]. Upon intertumoral injection, the LG precursor underwent a reversible change of gel-to-sol transition when the ambient temperature rose to 39 °C. The drug reservoir located in close proximity to the tumor site demonstrated persistent drug release, regulated by temperature fluctuations. This approach was subsequently validated for its ability to enhance the infiltration of lymphocytes into solid tumors of diverse origins. This approach effectively integrates the benefits of photothermal therapy and immunotherapy, capitalizing on the inherent low toxicity and biocompatibility of fundamental materials. There is less worry regarding its safety, and it has been observed that the innate immune system has the ability to protect against foreign substances, such as lipid-derived nanoparticles. These nanoparticles have the potential to activate the complement system through both the conventional and alternative pathways [119]. However, lipid-based biomaterials possess distinct characteristics that set them in cancer therapy. A recent study demonstrated the potential for drug delivery via the skin or the blood-brain barrier. The lipid-based nanotechnology, which is projected to account for more than 50% of all lipid-based nano formulations [121], will make more contributions in the near future.

In addition to the aforementioned common materials, it is worth noting that there are additional possibilities for the creation of nanoparticles. The exceptional biocompatibility and consistency batch-to-batch consistency of protein products suggest that they may be well-received for clinical translation [122]. Cai’s team took advantages of human serum albumin (HAS) to create nanoparticles, thereby capitalizing on its inherent benefits. HAS has been found to exhibit remarkable stability and solubility within living organisms. Previous studies have demonstrated its ability to selectively target and affect tumor cells, making it a promising candidate for drugs carrying [123]. In this study, the HAS was served as a nanochaperone for the delivery of interleukin-12 (IL-12), the molecule that would otherwise induce severe systemic proinflammatory toxicity when systemic administrated. The IL-12 ultimately facilitated the secretion of CCL5, CLL2, and CXCL10, leading to substantial proliferation of CD8+ CAR-Ts. Beyond that, Wang et al. fabricated a unique nanoparticle derived from yeast cell wall, taking into account the potential contribution of microbes in activating the innate immune system and enhancing the effectiveness of the adoptive immunological response, so bolstering the antitumor immune reaction. Significant tumor regression was seen in melanoma-bearing mice with the utilization of PD-L1 inhibition. Nevertheless, the advancement of microbial-based cancer therapy is hindered by the presence of dose-dependent side effects, such as systemic toxicity [124]. Irvine et al. developed protein nanogels, which were utilized for the delivery of IL-15 super-agonist. When conjugated with cell surface proteins, nanogels demonstrated the ability to unload drugs in a regulated manner upon receiving TCR signals during antigen recognition [125].

### Inorganic materials

Inorganic materials have made tremendous achievements recent years and are promising to distinguish themselves in drug delivery or other biomedicine for their special biochemical signatures.

Porous inorganic biomaterials have the satisfactory large surface area, stability in in vivo environment and diverse architectures. As opposed to common inorganic material, silica, calcium carbonate demonstrates great biocompatibility and biodegradability by imitating internal biominerals [126,127]. Further, CaCO_3_ is capable of carrying multiple drugs simultaneously, which was called “molecular cocktail” by researchers [128]. It is also found that Ca2+ could increase gene transfection efficacy when coprecipitated with DNA [129].

Graphene oxide is another attractive inorganic material. The 2-dimensional planar structure has won it considerable interest. Graphene-based structure is featured with unparalleled loading efficacy and acknowledged photosensitivity and conductivity, which guaranteed its position in material development [130,131].

Metal–organic frameworks (MOFs) are also burgeoning in drug delivery field. The scaffold possesses high storage capacity and readily manipulated for different functions. They could interplay with biological substance including bioactive cells. Zirconium MOFs have stood out amidst MOFs these days and are valued for both chemical and mechanical stability thanks to stronger Zr-carboxylate bonds and excellent porosity compared with remaining MOFs [132]. It is believed that MOFs will play a role in gene delivery in the near future.

### Scaffold-based systems

Since the first introduction in 1954, there has been a significant surge in research focused on the development of hydrogels. The hydrogel is well recognized as an exceptional biomaterial due to its numerous advantages. Firstly, hydrogel is a 3D structure comprised of hydrophilic polymers, which can be either of natural or synthetic origin. It has a high capacity for water absorption with ideal biodegradability, biocompatibility, and neglectable cellular toxicity. Secondly, it provides accurate and controlled release of substances, such as chemotherapy drugs or adjuvant agents (e.g., cytokines, antigens), in response to specific environmental trigger factors. These triggers can include external stimuli like mechanical forces, temperature, and magnetic fields, as well as internal stimuli such as pH, reactive oxygen concentration, and enzymatic activity. This phenomenon is sometimes referred to as its “reversible behavior”. In the context of hydrogels, it has been shown that the retention duration is extended, leading to an expansion of multifunctional therapeutic performance. This enhancement can be attributed to the distinctive material characteristics of hydrogels, which also contribute to improved mechanical qualities. Furthermore, hydrogel has a great degree of tenability in terms of sizes and delivery methods, which encompass injection, transdermal delivery, in situ implantation, and other approaches, thus reducing adverse effects associated with systemic administration. Ultimately, hydrogel is one of few materials capable of the 3D models applied in bioengineering. As one of paramount qualities making it indispensable, hydrogel 3D model could simulate the realistic TME while exhibiting traits, such as the stiffness and ductility, and environmental heterogeneity that closely resemble those of the TME. This could potentially make a significant contribution to the verification of safety for biomaterials relevant to contemporary cancer therapy [133–135].

Natural polymer hydrogel is predominantly beneficial due to its potential to safeguard cell viability while minimally impeding cell bioactivity. Simultaneously, they demonstrate the production of endogenous extracellular signals and ligands without the requirement for complex artificial integration. One of the obstacles that can be identified is the lack of diversity across batches, leading to a sense of dissatisfaction. Additionally, another challenge is the rather unpredictable nature of biodegradation [134].

Natural gellan gum (GG) was recently adopted as a novel approach for efficiently stimulating T cell subpopulations through antigen presentation. Derived from bacterial exopolysaccharide, GG has consistency across batches and biocompatibility. Prior studies mirrored that it had superior capability of cell capture, adhesion, and proliferation without expensive polymer modifications to improve cell entrapment, as compared to traditional hydrogel [136]. Recently, it has been reported the successful development of GG-based tailorable nanoparticles system for T cell activation. With specific ligands on its surface, these nanoparticles were found to generate large quantities of CD4^+^ regulatory T cells and inhibit T cells exhaustion in vitro. Due to its stability upon rehydration and enormous water uptake capacity, npGG particles have the potential to participate in downstream processes by absorbing biologically relevant molecules and facilitating their controlled in situ release. However, the logical relationship between these factors is unclear. This study presented a novel perspective on the utilization of hydrogels in the context of CAR-T therapy [26].

Another example of polysaccharide-based systems is the fucoidan-based complex coacervate-laden injectable hydrogel (FPC2-IG). Fucoidan belongs to glycosaminoglycan, a group of materials that have garnered significant attention in the field of protein delivery system. Glycosaminoglycan-based delivery systems exhibit notable efficacy in augmenting protein bioactivity. Fucoidan, owing to its remarkable protein binding capability, particularly in relation to interleukin-2 (IL-2), along with its low immunogenicity and natural abundance, demonstrates significant promise. Besides, the complex coacervate is a liquid–liquid phase separation phenomenon discovered in marine species, whose secretion of water-soluble polyelectrolytes formulate coacervates. It is the water-immiscibility, microencapsulation capability that contribute to high-effective delivery and sustained retention of uploaded protein in case of enzymatic degradation upon arrival of tumor sites. The researches selected poly-l-lysine, which has been approved by the U.S. Food and Drug Administration, as a positive electrolyte coupled with negatively charged FPC to form stable complex coacervate. The resulting layer was then embedded in a pH-regulated injectable hydrogel (FPC2-IG). To provide further clarification, the FPC2-IG-IL-2 treatment ultimately boosts the proliferation of cytotoxic lymphocytes and decreases the presence of myeloid populations by extending the duration of IL-2 release and modifying TME [137].

**Fig. 4.**
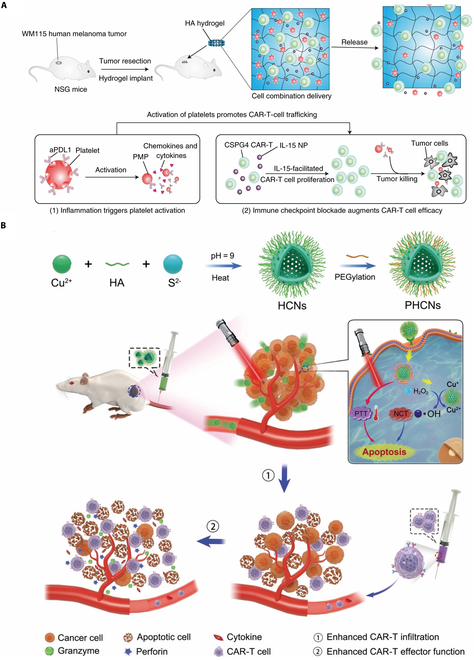
(A) Schematic of the tumor resection model and implantation of the engineered HA hydrogel, which was developed by a biodegradable hydrogel reservoir that encapsulates CAR-Ts targeting the human chondroitin sulfate proteoglycan 4. Copyright © 2021, Quanyin Hu et al., under exclusive license to Springer Nature Limited [138]. (B) A synergetic strategy by combination of nanozymes and CAR-Ts in solid tumors for enhanced infiltration and effector function of CAR-Ts. Copyright © 2021, Wiley [162].

The application of hyaluronic acid (HA) has also been documented. Gu et al. developed the crosslink procedure of hydrogel through ultraviolet, including an acrylate group into the hydrogel, as shown in Fig. 4A. The study involved the development of a product that combined IL-15 encapsulation with platelets conjugated with PD-L1 inhibitors. This product aimed to enhance the long-term storage of medications and improve the dispersion of CAR-Ts when implanted in the remaining cavity following tumor excision, with the objective of preventing tumor relapse [138].

Alginate is readily available in nature and possesses a consistent capacity for reproduction. It is produced by bacterial biosynthesis tends to exhibit superior quality. Additionally, it possesses excellent biocompatibility and has demonstrated biodegradation without causing any adverse effects on genetic material or cell viability, owing to its inert characteristics. The primary polymer structure exhibits a significant abundance of hydroxyl and carboxyl functional groups, which have garnered considerable interest among researchers in the field of biosynthesis due to their facile modifiability and hydrophilic nature. Alginate hydrogel exhibits both good oxidation tolerance and immune-stimulation. Thus, these materials are widely used in drug-delivery system establishment [139,140]. Chao et al. created a metformin-loaded alginate hydrogel which exerted positive effects on both TME regulation as well as CAR-T metabolism and activation [141]. Composite platform based on alginate hydrogel was also investigated to explore better way of in situ or ex vivo activation of CAR-Ts. Wang’s team designed an injectable microporous hydrogel. This innovates platform provided a storage solution for CAR-Ts, utilizing a nanoporous material matrix to reverse T cells activation signal. In the presence of T cells abundant in thiol groups, azido-functionalized alginate-based bulk-phase gel network can give a controlled release of microparticles that are bounded with anti-CD3 and anti-CD28 to effectively activate T cells in a responsive manner. Cost reduction related to cumbersome and error-prone manufacture procedure and allocation could be lower because such platform stored and sheltered the CAR-Ts prior to their delivery to the intended site, ensuring optimal therapy concentration within the tumor [142].

An injectable and photocurable hydrogel composed of gelatin methacryloyl was also documented in a scholarly study. The gelatin methacryloyl underwent scaffold formation with appropriate exposure to blue light. Within this scaffold, CAR-Ts had demonstrated a distinct distribution and maintained their biological activity within the TME. The procedure of curing solely necessitated photo-crosslinking for priming. It was relatively gentle and harmless processing means, requiring neither complex surgical procedures nor intricate environmental adjustments, and exhibiting a low tolerance to faults [31].

Regarding artificial synthesized hydrogel, have greater control over customizing its physical features and associated functionalities by using adjuvants, maintaining the batch-to-batch stability. Enzymolysis and recognition sites mimicking natural extracellular matrix could be secured through chemical synthesis [133,143].

Employed for persistent vaccine delivery, polymer-nanoparticle (PNP) hydrogels have been currently discovered to have potential application in CAR-T therapy. The injectable and self-assembled PNP were shown to induce a transitory inflammatory microenvironment, leading to improved proliferation and activation of immune cells. As shown in Fig. 4B, Zhu et al. described a synergetic strategy by combination of nanozymes and CAR-Ts in solid tumors to remodel TME. Researchers also found that both proximal and distal tumors exhibited satisfactory tumor regression after administration, suggesting its potent abscopal effect to eradicate inaccessible tumors. Subsequent observation revealed that PNP hydrogels could prevent the passive diffusion of cytokines while facilitating active motility of CAR-Ts. It could conclude that this technique exhibits great promises for future development [44]. By virtue of its excellent biocompatibility and adaptability, the collaboration between hydrogel and other materials holds significant potential for yielding unexpected gains. Hu et al. conducted a pioneering attempt to transform the live DCs into artificial APCs (aAPCs) which holds dominant responsibility for antigen-presenting and CAR-T activation. In this work, researcher opted for the utilization of a photo initiator known as 2-hydroxyl-4’-(2-hydroxyethoxy)-2-methylpropiophenone photoinitiator (I2959) and poly (ethylene glycol) diacrylate (PEG-DA) as the hydrogel materials to enhance direct membranous permeation in case of mechanical membrane disruption in original investigations. The hydrogel monomers underwent crosslinking upon exposure to UV radiation, forming the gelated DCs. The feasibility of storage by freezing and lyophilization and its off-the-shelf convenience was proved through a series of studies. The hydrogels were capable of loading customized antigen peptide and coupled with microparticles releasing immune-boosting cytokines. Moreover, researchers applied such methodology on autologous primary human DCs, and those constructions retained inceptive bioactivity and stability. The research opened up novel possibility for combination between natural and man-made hybrid material system [144].

Watson–Crick base pairing rules as underlying principle, the strict interaction nature of 4 nucleotides makes the programmable nanoscale assembly of 2D or 3D DNA structures possible. Under the guidance of Rothemund’s “scaffolded DNA origami” approach, it becomes possible to achieve the accurate fabrication of specified 3D structures using DNA and probably helps the large-scale production in the foreseeable future. Pure nucleic acid generated material lack of desirable electrical, mechanical, or catalytic properties. Therefore, chemical modification is commonly employed to introduce extra functionality [145–147]. Recently, scientists introduced such technology into CAR-T therapy augmentation. In an attempt to address the unfulfilled requirements for biocompatible conjugation platform that could integrate multiple biomolecules in harmony on surface, oligonucleotides were exploited to provide a solution. The brief synthetic DNA scaffolds was then immobilized on polymer particles fabricated with poly (lactic-co-glycolic acid) (PLGA) to form the biocompatible immune cell-engaging particles. Through the hybridization of cDNA strands, protein of variety was further coloaded and attached to respective scaffold population. Subsequent experiments confirmed that immune cell-engaging particles, a highly modularized platform, have multiple functions that are contingent upon the assembly of variant proteins. One notable example is its ability to present antigens in a manner that enables precise control over the activation of CAR-Ts, like an AND-gate mechanism. It is believed that such modular and versatile platform have a significant impact for immune regulation in clinical practice [148].

**Fig. 5.**
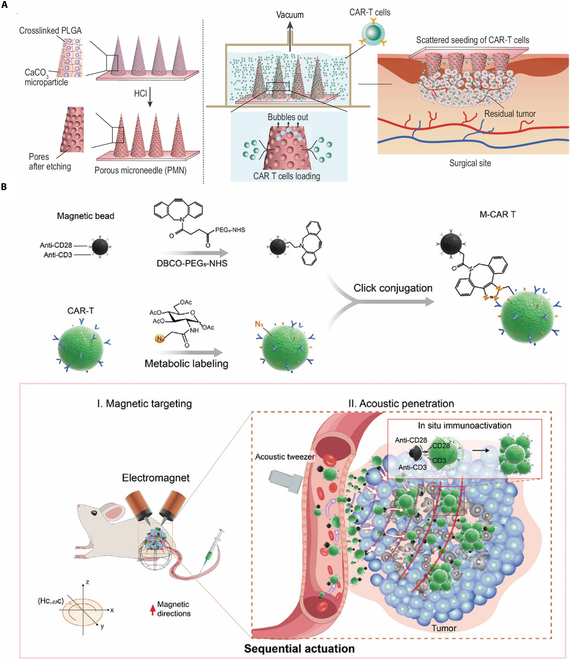
(A) Characterization of PMN patch for CAR-T loading and delivery. Copyright © 2021, © Li, Hongjun; Wang, Zejun 2021. Published by Oxford University Press on behalf of China Science Publishing & Media Ltd [151]. (B) A CAR-T-based live microrobot (M-CAR T) created by decorating CAR T with immunomagnetic beads using click conjugation. Copyright © 2023, Wiley [159].

Comprised of series of microscale needles, microneedle emerge as promising drug delivery platform. Microneedles have been available in several designs including solid, hollow, dissolving and drug reservoir, with each shape being tailored for varied delivery principals. An instance of a dissolving microneedle can be manufactured using biodegradable materials such as PVP. Drugs are capable to be continually released in the planting area following the gradual dissolution of needles to increase bioactive concentration. In addition to its local-controllable flexibility, microneedles are malleable for stimuli-responsive potential by means of utilizing specific materials. According to different biochemical and physical signal condition, microneedle exhibits the ability to integrate environmental analysis and supportive drugs delivery into 1 structure. Compared with other drugs delivery platforms, microneedles handle drugs fluctuating in size and species over a wider range of medicines varying in size and species. The possible cargo options encompass small molecules, macromolecules, nanoparticles or immune cells, which can be utilized for various purposes such as anti-inflammation, antibacteria, immune regulation, or tissue targeting. Additionally, the sharp and minuscule tip of needle allows it to bypass external impeditive barrier for drugs to penetrate, while minimizing the associated pain, hence enhancing patient compliance. Yet, it is of note that the balance between porosity and critical parameters such as mechanical force and cargo capacity must be carefully evaluated [103,149,150]. Gu’s team developed a polymer porous microneedle (PMN) patch in order to showcase a CAR-T delivery vehicle that is a multipoint, scattered, yet evenly distributed administration, as shown in Fig. 5A [151].

In present studies, PLGA was employed as a structural material to provide adequate stiffness for the insertion of the therapeutic agent into the tumor. The pores were created after neutralization reaction to accommodate the CAR-T therapy. Through experimental results, it was found that PMN-mediated transport caused homologous mapping while intra-tumor injection led to cell restriction. The PMN patch also maintained the cell viability by sheltering CAR-T within the pores. Additionally, it effectively penetrated the external physical barrier of solid tumors and enhanced the distribution of cells [151].

Nitinol has a rich historical background in its extensive utilization as an implant, particularly in cardiovascular [152] and other related domains, owing to its exceptional biocompatibility. In recent years, its application has been extended to immune cell therapy. Stephan’s team fabricated micropatterned nickel titanium (also known as nitinol) using magnetron sputtering technique. The nitinol material exhibits a well-defined geometry with a high resolution at micrometre scale. This characteristic enhances the predictability of cargo delivery kinetics when compared to the random pore network found in polymer-based scaffolds. In addition, it demonstrates superior space utilization rate and elasticity, stability, and shape memory. All of these satisfied the requirements for preventing blockage of the implanted lumen, taking into account external compressive force or unknown luminal abnormalities. In this research, the fibrin layer of nitinol with T cell stimulant signal molecules was involved when T cell infiltrated into engineered micropatterns. The thin film functioned as both the catalyst for CAR-T multiplication and the carrier for targeted transportation. Additionally, it acted as a tumor stent, impeding tumor growth and prolonging overall survival. Nevertheless, a dual-sided dilemma arose regarding the nondegradability of nitinol [153].

Mechanical changes in tissues influence immune cells, tuning their effector functions, with the potential to promote both adaptive and maladaptive responses [154]. Immune cells exhibited stronger signaling responses and cytokine secretion on stiff surfaces than they did on softer surfaces [155]. The mechanical properties of some materials can be adjusted by varying the number of monomers or the degree of crosslinking, giving us the ability to manipulate the mechanical properties of the extracellular matrix [156]. Thus, through adjusting stiffness, we are capable to modulate immune cell activation [157].

There continue to exist a wide range of efficacy-enhancing methods that have been derived from diverse ideas an illustrative instance would be the utilization of a vaccine-like enhancer for CAR-T therapy. The researchers developed a novel class of ligands, known as amphiphile CAR-T ligands (amph-ligands), which can effectively target lymph nodes through conjugation with albumin-binding phospholipid polymers. Upon the entrance of lymph nodes, the ligand is transferred to APCs, triggering the secretion of cytokines which subsequently prime the T cells that possess the predetermined and engineered CAR on their ligands [158]. Moreover, as shown in Fig. 5B, Tang et al. created a CAR-T-based live microrobot (M-CAR T) by decorating CAR T with immunomagnetic beads using click conjugation [159]. There remain significant opportunities for further advancement in the development of biomaterials for CAR-T therapy, as a substantial number of these materials have not yet undergone clinical trials or achieved compliance with the good manufacturing practice standard. To yet, the valuable biosynthetic properties exhibited by many materials in medical research have not been effectively translated into immune therapy, specifically CAR-T therapy.

## Advancements

In recent years, propelled by remarkable strides in biomaterials and cell engineering technology, investigators have increasingly directed their attention toward harnessing biomaterials to augment the effectiveness of cellular immunotherapy. These endeavors encompass a range of strategies, which include mitigating immune evasion, achieving precise, targeted cell delivery, bolstering the survival of engineered T cells, and even orchestrating synergistic combination therapies.

**Fig. 6.**
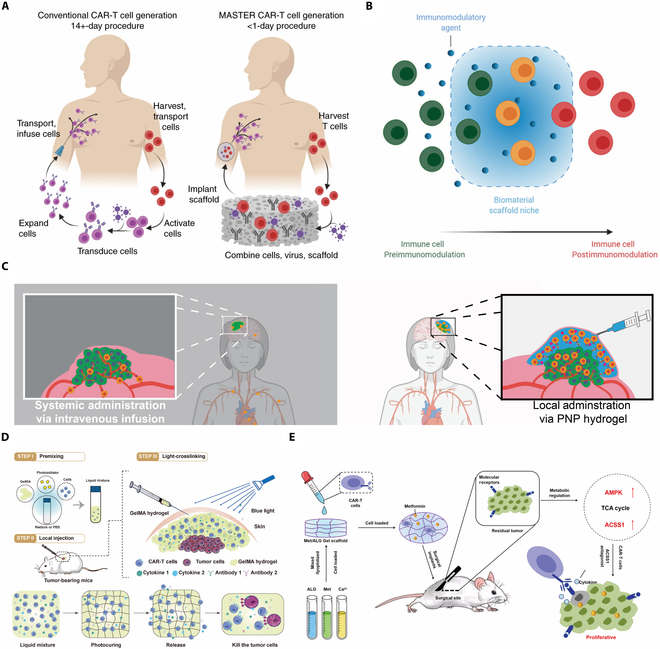
(A) Rapid MASTER-mediated CAR-T generation and therapy compared to conventional CAR-T therapy. Copyright © 2022, Pritha Agarwalla et al., under exclusive license to Springer Nature America, Inc [160]. (B) Biomaterial scaffold niche in the area of cancer immunotherapy. Copyright © 2020 American Chemical Society [161]. (C) Schematic illustration demonstrating our proposed delivery method for CAR-Ts to solid tumors compared to tradition intravenous approaches. Copyright © 2022 Abigail K. Grosskopf et al. [32]. (D) Schematic of the injectable CAR-T delivery system. © 2022 Elsevier Ltd [31]. (E) Schematic illustration of tumor resection and implantation of CAR-T@Met/gel. © 2023 Elsevier Ltd [141].

Agarwalla et al. described an implantable Multifunctional Alginate Scaffold for T Cell Engineering and Release (MASTER) that streamlines in vivo CAR-T manufacturing and control distal tumor growth in a mouse xenograft model of lymphoma, as shown in Fig. 6A [160]. This nuanced approach not only holds the promise of optimizing therapeutic efficacy but also attenuating systemic exposure, thereby presenting a noteworthy advancement in the field of immunotherapeutic intervention, as shown in Fig. 6B [161]. Despite the notable achievements of CAR-T therapy in the management of hematologic malignancies, its efficacy in treating solid tumors remains limited. The advancements of biomaterials have allowed CAR-T therapy to emerge in the treatment of solid tumors.

Grosskopf et al. developed a simple-to-implement injectable hydrogel for the controlled co-delivery of CAR-Ts and stimulatory cytokines that improve efficacy of solid tumors therapy (Fig. 6C) [32]. Furthmore, Zhou et al. engineered an injectable CAR-T gelatin methacryloyl hydrogel drug delivery system, which was abbreviated as i-GMD. Impressively, this construct exhibited heightened antitumor efficacy, yielding a substantial increase in the survival rates of mice bearing melanoma. Cumulatively, these efforts underscore the potential of biomaterial scaffolds to function as conduits for delivering immunomodulators or immune cells (Fig. 6D) [31].

In addition, biomaterials, as a widely employed drug delivery approach, have shown their distinct advantages in incorporating CAR-T therapy. A pertinent instance in this realm is provided by the research conducted by Yu Chao et al. The researchers developed the CAR-T-Met@gel system, a hydrogel scaffold proficient in codelivering both metformin and CAR-Ts (Fig. 6E) [141]. By incorporating CAR-Ts into a freeze-dried alginate hydrogel harbouring metformin, a platform was established wherein therapeutic agents and cells were smoothly merged. The effectiveness of this approach was additionally corroborated through rigorous in vivo experimentation on murine gastric cancer models. Noteworthy achievements included not only curtailing postoperative tumor recurrence but also impeding the growth of distant neoplasms.

## Conclusions and Perspective

Cellular immunotherapy represents a highly promising strategy for treatment of tumor, wherein activated immune cells are employed to identify and eliminate specific tumor cells with better efficacy compared to traditional treatment modalities. Nevertheless, the clinical implementation of cellular immunotherapy is hindered by the notable problems of low response rates and adverse effects. Furthermore, its efficacy in treating solid tumors has been suboptimal.

Biomaterials confer significant advantageous applications in the field of cellular immunotherapy. Within this domain, biomaterials demonstrate the potential to enhance drug delivery precision, maintain cellular vitality, enable controlled release dynamics, possess immunomodulatory prowess, and boost treatment resilience. Collectively, these attributes synergistically amplify the efficacy and viability of cellular immunotherapy. Biomaterials serve as adept vehicles for ferrying active constituents, such as immune cells, cytokines, or pharmaceutical agents, efficiently transporting them to targeted treatment sites. This orchestrated delivery augments therapeutic potency, which is an important factor of paramount significance in addressing solid tumors. In addition, biomaterials furnish a conducive environment that fosters cellular adhesion, proliferation, and differentiation. This nurturing environment facilitates the sustained cellular vitality, hence optimizing therapeutic impact. By establishing a propitious microenvironment, biomaterials adeptly extend the durability and effectiveness of cellular immunotherapy, sustaining its potency within the body over an extended period of time. By leveraging techniques that encompass shape molding and bio-3D printing, diverse treatment paradigms can be tailored to individual patients, thereby ushering in personalized therapeutic strategies with enhanced efficacy.

## References

[1] Zhang Y, Zhang Z. The history and advances in cancer immunotherapy: Understanding the characteristics of tumor-infiltrating immune cells and their therapeutic implications. Cell Mol Immunol. 2020;17(8):807–821. 32612154 10.1038/s41423-020-0488-6 PMC7395159

[2] Finck AV, Blanchard T, Roselle CP, Golinelli G, June CH. Engineered cellular immunotherapies in cancer and beyond. Nat Med. 2022;28(6622):678–689. 35440724 10.1038/s41591-022-01765-8 PMC9305718

[3] Mullard A. FDA approves fourth CAR-T cell therapy. Nat Rev Drug Discov. 2021;20:166–166. 10.1038/d41573-021-00031-9 33574586

[4] Guo J, Tang Q. Recent updates on chimeric antigen receptor T cell therapy for hepatocellular carcinoma. Cancer Gene Ther. 2021;28(10–11):1075–1087. 33500535 10.1038/s41417-020-00259-4

[5] Sterner RC, Sterner RM. CAR-T cell therapy: Current limitations and potential strategies, blood. Cancer J. 2021;11:69. 10.1038/s41408-021-00459-7 PMC8024391 33824268

[6] Sheykhhasan M, Manoochehri H, Dama P. Use of CAR T-cell for acute lymphoblastic leukemia (ALL) treatment: A review study. Cancer Gene Ther. 2022;29(8–9):1080–1096. 34987176 10.1038/s41417-021-00418-1 PMC9395272

[7] Wei J, Guo Y, Wang Y, Wu Z, Bo J, Zhang B, Zhu J, Han W. Clinical development of CAR T cell therapy in China: 2020 update. Cell Mol Immunol. 18(2021):792–804. 32999455 10.1038/s41423-020-00555-x PMC8115146

[8] Govers C, Sebestyén Z, Coccoris M, Willemsen RA, Debets R. T cell receptor gene therapy: Strategies for optimizing transgenic TCR pairing. Trends Mol Med. 2010;16(2):77–87. 20122868 10.1016/j.molmed.2009.12.004

[9] Baulu E, Gardet C, Chuvin N, Depil S. TCR-engineered T cell therapy in solid tumors: State of the art and perspectives. Sci Adv. 2023;9(7):eadf3700. 36791198 10.1126/sciadv.adf3700 PMC9931212

[10] Morotti M, Albukhari A, Alsaadi A, Artibani M, Brenton JD, Curbishley SM, Dong T, Dustin ML, Hu Z, McGranahan N, et al. Promises and challenges of adoptive T-cell therapies for solid tumours. Br J Cancer. 2021;124(11):1759–1776. 33782566 10.1038/s41416-021-01353-6 PMC8144577

[11] Xie G, Dong H, Liang Y, Ham JD, Rizwan R, Chen J. CAR-NK cells: A promising cellular immunotherapy for cancer. EBioMedicine. 2020;59: Article 102975. 32853984 10.1016/j.ebiom.2020.102975 PMC7452675

[12] El-Mayta R, Zhang Z, Hamilton AG, Mitchell MJ. Delivery technologies to engineer natural killer cells for cancer immunotherapy. Cancer Gene Ther. 2021;28(9):947–959. 33888870 10.1038/s41417-021-00336-2

[13] Albinger N, Hartmann J, Ullrich E. Current status and perspective of CAR-T and CAR-NK cell therapy trials in Germany. Gene Ther. 2021;28(9):513–527. 33753909 10.1038/s41434-021-00246-w PMC8455322

[14] Fares J, Davis ZB, Rechberger JS, Toll SA, Schwartz JD, Daniels DJ, Miller JS, Khatua S. Advances in NK cell therapy for brain tumors. NPJ Precis Oncol. 2023;7(1):17. 36792722 10.1038/s41698-023-00356-1 PMC9932101

[15] Daher M, Melo Garcia L, Li Y, Rezvani K. CAR-NK cells: The next wave of cellular therapy for cancer. Clin Transl Immunology. 2021;10(4): Article e1274. 33959279 10.1002/cti2.1274 PMC8080297

[16] Liu E, Marin D, Banerjee P, Macapinlac HA, Thompson P, Basar R, Nassif Kerbauy L, Overman B, Thall P, Kaplan M, et al. Use of CAR-transduced natural killer cells in CD19-positive lymphoid tumors. New Engl J Med. 2020;382(6):545–553. 32023374 10.1056/NEJMoa1910607 PMC7101242

[17] Denman CJ, Senyukov VV, Somanchi SS, Phatarpekar PV, Kopp LM, Johnson JL, Singh H, Hurton L, Maiti SN, Huls MH, et al. Membrane-bound IL-21 promotes sustained ex vivo proliferation of human natural killer cells. PLOS ONE. 2012;7(1): Article e30264. 22279576 10.1371/journal.pone.0030264 PMC3261192

[18] Martins F, Sofiya L, Sykiotis GP, Lamine F, Maillard M, Fraga M, Shabafrouz K, Ribi C, Cairoli A, Guex-Crosier Y, et al. Adverse effects of immune-checkpoint inhibitors: Epidemiology, management and surveillance. Nat Rev Clin Oncol. 2019;16(9):563–580. 31092901 10.1038/s41571-019-0218-0

[19] Okwundu N, Grossman D, Hu-Lieskovan S, Grossmann KF, Swami U. The dark side of immunotherapy. Ann Transl Med. 2021;9(12):1041–1041. 34277841 10.21037/atm-20-4750 PMC8267325

[20] Albelda SM. CAR T cell therapy for patients with solid tumours: Key lessons to learn and unlearn. Nat Rev Clin Oncol. 2023;21(1):47–66. 37904019 10.1038/s41571-023-00832-4

[21] Flugel CL, Majzner RG, Krenciute G, Dotti G, Riddell SR, Wagner DL, Abou-el-Enein M. Overcoming on-target, off-tumour toxicity of CAR T cell therapy for solid tumours. Nat Rev Clin Oncol. 2023;20(1):49–62. 36418477 10.1038/s41571-022-00704-3 PMC10278599

[22] Agarwalla P, Ogunnaike EA, Ahn S, Ligler FS, Dotti G, Brudno Y. Scaffold-mediated static transduction of T cells for CAR-T cell therapy. Adv Healthc Mater. 2020;9(14):e2000275. 32592454 10.1002/adhm.202000275 PMC7518635

[23] Rauch-Wirth L, Renner A, Kaygisiz K, Weil T, Zimmermann L, Rodriguez-Alfonso AA, Schütz D, Wiese S, Ständker L, Weil T, et al. Optimized peptide nanofibrils as efficient transduction enhancers for in vitro and ex vivo gene transfer. Front Immunol. 2023;14:1270243. 38022685 10.3389/fimmu.2023.1270243 PMC10666768

[24] Ma B, Liu F, Zhang S, Duan J, Kong Y, Li Z, Tang D, Wang W, Ge S, Tang W, et al. Two-photon fluorescent polydopamine nanodots for CAR-T cell function verification and tumor cell/tissue detection. J Mater Chem B. 2018;6:6459–6467. 32254653 10.1039/c8tb01930d

[25] Kiru L, Zlitni A, Tousley AM, Dalton GN, Wu W, Lafortune F, Liu A, Cunanan KM, Nejadnik H, Sulchek T, et al. In vivo imaging of nanoparticle-labeled CAR T cells. Proc Natl Acad Sci USA. 2022;119(6): Article e2102363119. 35101971 10.1073/pnas.2102363119 PMC8832996

[26] Rodrigues DB, Moreira HR, Cerqueira MT, Marques AP, Castro AG, Reis RL, Pirraco RP. Highly tailorable gellan gum nanoparticles as a platform for the development of T cell activator systems. Biomater Res. 2022;26(1):48. 36180901 10.1186/s40824-022-00297-z PMC9523970

[27] Wang H, Mooney DJ. Biomaterial-assisted targeted modulation of immune cells in cancer treatment. Nat Mater. 2018;17(9):761–772. 30104668 10.1038/s41563-018-0147-9

[28] Wu S-Y, Ji F, Xu B, Wu F-G. Delivering CAR-T cells into solid tumors via hydrogels. MedComm–Oncology. 2023;2(2): Article e40.

[29] Engineering CART. Cells with biomaterials. Cancer Discov. 2017;7:656–657. 10.1158/2159-8290.CD-NB2017-068 28500027

[30] Zhang D, Li Q, Chen X, Nie X, Xue F, Xu W, Luan Y. An injectable hydrogel to modulate T cells for cancer immunotherapy. Small. 2022;18(32):2202663. 10.1002/smll.202202663 35843879

[31] Zhou W, Lei S, Liu M, Li D, Huang Y, Hu X, Yang J, Li J, Fu M, Zhang M, et al. Injectable and photocurable CAR-T cell formulation enhances the anti-tumor activity to melanoma in mice. Biomaterials. 2022;291: Article 121872. 36323072 10.1016/j.biomaterials.2022.121872

[32] Grosskopf AK, Labanieh L, Klysz DD, Roth GA, Xu P, Adebowale O, Gale EC, Jons CK, Klich JH, Yan J, et al. Delivery of CAR-T cells in a transient injectable stimulatory hydrogel niche improves treatment of solid tumors. Sci Adv. 2022;8(14):eabn8264. 35394838 10.1126/sciadv.abn8264 PMC8993118

[33] Jie J, Mao D, Cao J, Feng P, Yang P. Customized multifunctional peptide hydrogel scaffolds for CAR-T-cell rapid proliferation and solid tumor immunotherapy. ACS Appl Mater Interfaces. 2022;14(33):37514–37527. 35944246 10.1021/acsami.2c10727

[34] Yang F, Shi K, Jia Y, Hao Y, Peng J, Qian Z. Advanced biomaterials for cancer immunotherapy. Acta Pharmacol Sin. 2020;41(7):911–927. 32123302 10.1038/s41401-020-0372-z PMC7468530

[35] Wang X, Rivière I. Clinical manufacturing of CAR T cells: Foundation of a promising therapy. Mol Ther Oncolytics. 2016;3:16015. 27347557 10.1038/mto.2016.15 PMC4909095

[36] Blache U, Popp G, Dünkel A, Koehl U, Fricke S. Potential solutions for manufacture of CAR T cells in cancer immunotherapy. Nat Commun. 2022;13(1):5225. 36064867 10.1038/s41467-022-32866-0 PMC9445013

[37] Abou-el-Enein M, Elsallab M, Feldman SA, Fesnak AD, Heslop HE, Marks P, Till BG, Bauer G, Savoldo B. Scalable manufacturing of CAR T cells for cancer immunotherapy, blood cancer. Discovery. 2021;2(5):408–422. 10.1158/2643-3230.BCD-21-0084 PMC8462122 34568831

[38] Porter DL, Hwang W-T, Frey NV, Lacey SF, Shaw PA, Loren AW, Bagg A, Marcucci KT, Shen A, Gonzalez V, et al. Chimeric antigen receptor T cells persist and induce sustained remissions in relapsed refractory chronic lymphocytic leukemia. Sci Transl Med. 2015;7(303):303ra139. 10.1126/scitranslmed.aac5415 PMC5909068 26333935

[39] Rosenberg SA, Restifo NP. Adoptive cell transfer as personalized immunotherapy for human cancer. Science. 2015;348(6230):62–68. 25838374 10.1126/science.aaa4967 PMC6295668

[40] Bischofberger M, Iacovache I, Gisou van der Goot F. Pathogenic pore-forming proteins: Function and host response. Cell Host Microbe. 2012;12(3):266–275. 22980324 10.1016/j.chom.2012.08.005

[41] Efficient genetic modification of murine dendritic cells by electroporation with mRNA | Cancer Gene Therapy, (n.d.). [accessed 22 September 2023] https://www.nature.com/articles/7700499 10.1038/sj.cgt.7700499 12189529

[42] Scherer F, Anton M, Schillinger U, Henke J, Bergemann C, Krüger A, Gänsbacher B, Plank C. Magnetofection: Enhancing and targeting gene delivery by magnetic force in vitro and in vivo. Gene Ther. 2002;9(2):102–109. 11857068 10.1038/sj.gt.3301624

[43] Xiong R, Raemdonck K, Peynshaert K, Lentacker I, De Cock I, Demeester J, De Smedt SC, Skirtach AG, Braeckmans K. Comparison of gold nanoparticle mediated photoporation: Vapor nanobubbles outperform direct heating for delivering macromolecules in live cells. ACS Nano. 2014;8(6):6288–6296. 24870061 10.1021/nn5017742

[44] Wang Y, Yang Y, Yan L, Kwok SY, Li W, Wang Z, Zhu X, Zhu G, Zhang W, Chen X, et al. Poking cells for efficient vector-free intracellular delivery. Nat Commun. 2014;5:4466. 25072981 10.1038/ncomms5466

[45] Sharei A, Zoldan J, Adamo A, Sim WY, Cho N, Jackson E, Mao S, Schneider S, Han M-J, Lytton-Jean A, et al. A vector-free microfluidic platform for intracellular delivery. Proc Natl Acad Sci USA. 2013;110:2082–2087. 23341631 10.1073/pnas.1218705110 PMC3568376

[46] Barber MA. A technic for the inoculation of bacteria and other substances into living cells. J Infect Dis. 1911;8(3):348–360.

[47] Fechheimer M, Boylan JF, Parker S, Sisken JE, Patel GL, Zimmer SG. Transfection of mammalian cells with plasmid DNA by scrape loading and sonication loading. Proc Natl Acad Sci USA. 1987;84(23):8463–8467. 2446324 10.1073/pnas.84.23.8463 PMC299564

[48] Shi J, Ma Y, Zhu J, Chen Y, Sun Y, Yao Y, Yang Z, Xie J. A review on electroporation-based intracellular delivery. Molecules. 2018;23(11):3044. 30469344 10.3390/molecules23113044 PMC6278265

[49] Sherba JJ, Hogquist S, Lin H, Shan JW, Shreiber DI, Zahn JD. The effects of electroporation buffer composition on cell viability and electro-transfection efficiency. Sci Rep. 2020;10:3053. 32080269 10.1038/s41598-020-59790-x PMC7033148

[50] Membrane electroporation theories: a review | SpringerLink, (n.d.). [accessed 22 September 2023] https://link.springer.com/article/10.1007/s11517-005-0020-2

[51] Zhang Z, Zheng T, Zhu R. Single-cell individualized electroporation with real-time impedance monitoring using a microelectrode array chip. Microsyst Nanoeng. 2020;6:81. 34567691 10.1038/s41378-020-00196-0 PMC8433324

[52] Lanciano S, Cristofari G. Measuring and interpreting transposable element expression. Nat Rev Genet. 2020;21:721–736. 32576954 10.1038/s41576-020-0251-y

[53] Amberger M, Ivics Z. Latest advances for the sleeping beauty transposon system: 23 years of insomnia but prettier than ever. BioEssays. 2020;42(11):e2000136. 32939778 10.1002/bies.202000136

[54] Cain AK, Barquist L, Goodman AL, Paulsen IT, Parkhill J, van Opijnen T. A decade of advances in transposon-insertion sequencing. Nat Rev Genet. 2020;21(9):526–540. 32533119 10.1038/s41576-020-0244-x PMC7291929

[55] Ye L, Lam SZ, Yang L, Suzuki K, Zou Y, Lin Q, Zhang Y, Clark P, Peng L, Chen S. AAV-mediated delivery of a Sleeping Beauty transposon and an mRNA-encoded transposase for the engineering of therapeutic immune cells. Nat Biomed Eng. 2023;8(2):132–148. 37430157 10.1038/s41551-023-01058-6 PMC11320892

[56] Li Y, Li L, Chen Z-N, Gao G, Yao R, Sun W. Engineering-derived approaches for iPSC preparation, expansion, differentiation and applications. Biofabrication. 2017;9(3): Article 032001. 28759433 10.1088/1758-5090/aa7e9a

[57] Jiang Z, Han Y, Cao X. Induced pluripotent stem cell (iPSCs) and their application in immunotherapy. Cell Mol Immunol. 2014;11(1):17–24. 24336163 10.1038/cmi.2013.62 PMC4002145

[58] Li W, Ding S. Small molecules that modulate embryonic stem cell fate and somatic cell reprogramming. Trends Pharmacol Sci. 2010;31(1):36–45. 19896224 10.1016/j.tips.2009.10.002

[59] Themeli M, Kloss CC, Ciriello G, Fedorov VD, Perna F, Gonen M, Sadelain M. Generation of tumor-targeted human T lymphocytes from induced pluripotent stem cells for cancer therapy. Nat Biotechnol. 2013;31(10):928–933. 23934177 10.1038/nbt.2678 PMC5722218

[60] Zhang L, Tian L, Dai X, Yu H, Wang J, Lei A, Zhu M, Xu J, Zhao W, Zhu Y, et al. Pluripotent stem cell-derived CAR-macrophage cells with antigen-dependent anti-cancer cell functions. J Hematol Oncol. 2020;13(1):153. 33176869 10.1186/s13045-020-00983-2 PMC7656711

[61] Tang SY, Zha S, Du Z, Zeng J, Zhu D, Luo Y, Wang S. Targeted integration of EpCAM-specific CAR in human induced pluripotent stem cells and their differentiation into NK cells. Stem Cell Res Ther. 2021;12(1):580. 34802459 10.1186/s13287-021-02648-4 PMC8607711

[62] Li Y, Hermanson DL, Moriarity BS, Kaufman DS. Human iPSC-derived natural killer cells engineered with chimeric antigen receptors enhance anti-tumor activity. Cell Stem Cell. 2018;23(2):181–192.e5. 30082067 10.1016/j.stem.2018.06.002 PMC6084450

[63] Lin X, Sun Y, Dong X, Liu Z, Sugimura R, Xie G. IPSC-derived CAR-NK cells for cancer immunotherapy. Biomed Pharmacother. 2023;165: Article 115123. 37406511 10.1016/j.biopha.2023.115123

[64] Shimasaki N, Shimizu E, Nakamura Y, Iguchi H, Ueda A, Umekage M, Haneda S, Mazda O. Size control of induced pluripotent stem cells colonies in two-dimensional culture for differentiation into functional monocyte-like cells. Cytotherapy. 2023;25(12):1338–1348. 37676216 10.1016/j.jcyt.2023.08.002

[65] Querques I, Mades A, Zuliani C, Miskey C, Alb M, Grueso E, Machwirth M, Rausch T, Einsele H, Ivics Z, et al. A highly soluble Sleeping Beauty transposase improves control of gene insertion. Nat Biotechnol. 2019;37(12):1502–1512. 31685959 10.1038/s41587-019-0291-z PMC6894935

[66] Dai X, Park JJ, Du Y, Kim HR, Wang G, Errami Y, Chen S. One-step generation of modular CAR-T cells with AAV–Cpf1. Nat Methods. 2019;16(3):247–254. 30804551 10.1038/s41592-019-0329-7 PMC6519746

[67] Irving M, Lanitis E, Migliorini D, Ivics Z, Guedan S. Choosing the right tool for genetic Engineering: Clinical lessons from chimeric antigen receptor-T cells. Hum Gene Ther. 2021;32(19–20):1044–1058. 34662233 10.1089/hum.2021.173 PMC8697565

[68] Batista Napotnik T, Polajžer T, Miklavčič D. Cell death due to electroporation–A review. Bioelectrochemistry. 2021;141: Article 107871. 34147013 10.1016/j.bioelechem.2021.107871

[69] Xiong R, Hua D, Van Hoeck J, Berdecka D, Léger L, De Munter S, Fraire JC, Raes L, Harizaj A, Sauvage F, et al. Photothermal nanofibres enable safe engineering of therapeutic cells. Nat Nanotechnol. 2021;16(11):1281–1291. 34675410 10.1038/s41565-021-00976-3 PMC7612007

[70] Ramon J, Xiong R, De Smedt SC, Raemdonck K, Braeckmans K. Vapor nanobubble-mediated photoporation constitutes a versatile intracellular delivery technology. Curr Opin Colloid Interface Sci. 2021;54: Article 101453.

[71] Yang W, Liang H, Ma S, Wang D, Huang J. Gold nanoparticle based photothermal therapy: Development and application for effective cancer treatment. Sustain Mater Technol. 2019;22: Article e00109.

[72] Teirlinck E, Xiong R, Brans T, Forier K, Fraire J, Van Acker H, Matthijs N, De Rycke R, De Smedt SC, Coenye T, et al. Laser-induced vapour nanobubbles improve drug diffusion and efficiency in bacterial biofilms. Nat Commun. 2018;9(1):4518–4512. 30375378 10.1038/s41467-018-06884-w PMC6207769

[73] Wayteck L, Xiong R, Braeckmans K, De Smedt SC, Raemdonck K. Comparing photoporation and nucleofection for delivery of small interfering RNA to cytotoxic T cells. J Control Release. 2017;267:154–162. 28778478 10.1016/j.jconrel.2017.08.002

[74] Harizaj A, Wels M, Raes L, Stremersch S, Goetgeluk G, Brans T, Vandekerckhove B, Sauvage F, De Smedt SC, Lentacker I, et al. Photoporation with biodegradable polydopamine nanosensitizers enables safe and efficient delivery of mRNA in human T cells. Adv Funct Mater. 2021;31(28):2102472.

[75] Hur J, Chung AJ. Microfluidic and nanofluidic intracellular delivery. Adv Sci. 2021;8(15):2004595. 10.1002/advs.202004595 PMC8336510 34096197

[76] Mykhaylyk O, Antequera YS, Vlaskou D, Plank C. Generation of magnetic nonviral gene transfer agents and magnetofection in vitro. Nat Protoc. 2007;2(10):2391–2411. 17947981 10.1038/nprot.2007.352

[77] Yang W, Tang Q, Bai Y, Wang K, Dong X, Li Y, Fang M. Bacterial magnetic particles-polyethylenimine vectors deliver target genes into multiple cell types with a high efficiency and low toxicity. Appl Microbiol Biotechnol. 2020;104:6799–6812. 32548689 10.1007/s00253-020-10729-2

[78] Labanieh L, Majzner RG, Mackall CL. Programming CAR-T cells to kill cancer. Nat. Biomed. Eng. 2018;2(6):377–391. 31011197 10.1038/s41551-018-0235-9

[79] Santiago-Ortiz JL, Schaffer DV. Adeno-associated virus (AAV) vectors in cancer gene therapy. J Control Release. 2016;240:287–301. 26796040 10.1016/j.jconrel.2016.01.001 PMC4940329

[80] Nyberg WA, Ark J, A. To, Clouden S, Reeder G, Muldoon JJ, Chung J-Y, Xie WH, Allain V, Steinhart Z, et al. An evolved AAV variant enables efficient genetic engineering of murine T cells. Cell. 2023;186(2):446–460.e19. 36638795 10.1016/j.cell.2022.12.022 PMC10540678

[81] Fraietta JA, Nobles CL, Sammons MA, Lundh S, Carty SA, Reich TJ, Cogdill AP, Morrissette JJD, DeNizio JE, Reddy S, et al. Disruption of TET2 promotes the therapeutic efficacy of CD19-targeted T cells. Nature. 2018;558(7709):307–312. 29849141 10.1038/s41586-018-0178-z PMC6320248

[82] Ekladious I, Colson YL, Grinstaff MW. Polymer–drug conjugate therapeutics: Advances, insights and prospects. Nat Rev Drug Discov. 2019;18(4):273–294. 30542076 10.1038/s41573-018-0005-0 PMC12032968

[83] Teo PY, Yang C, Whilding LM, Parente-Pereira AC, Maher J, George AJT, Hedrick JL, Yang YY, Ghaem-Maghami S. Ovarian cancer immunotherapy using PD-L1 siRNA targeted delivery from folic acid-functionalized polyethylenimine: Strategies to enhance T cell killing. Adv Healthc Mater. 2015;4(8):1180–1189. 25866054 10.1002/adhm.201500089

[84] Lin S, Du F, Wang Y, Ji S, Liang D, Yu L, Li Z. An acid-labile block copolymer of PDMAEMA and PEG as potential carrier for intelligent gene delivery systems. Biomacromolecules. 2008;9(1):109–115. 18088093 10.1021/bm7008747

[85] Yu Q, Zhang M, Chen Y, Chen X, Shi S, Sun K, Ye R, Zheng Y, Chen Y, Xu Y, et al. Self-assembled nanoparticles prepared from low-molecular-weight PEI and low-generation PAMAM for EGFRvIII-chimeric antigen receptor gene loading and T-cell transient modification. Int J Nanomedicine. 2020;15:483–495. 32158206 10.2147/IJN.S229858 PMC6986680

[86] Grünwald GK, Vetter A, Klutz K, Willhauck MJ, Schwenk N, Senekowitsch-Schmidtke R, Schwaiger M, Zach C, Wagner E, Göke B, et al. Systemic image-guided liver cancer radiovirotherapy using dendrimer-coated adenovirus encoding the sodium iodide symporter as theranostic gene. J Nucl Med. 2013;54(8):1450–1457. 23843567 10.2967/jnumed.112.115493

[87] Fornaguera C, Castells-Sala C, Lázaro MA, Cascante A, Borrós S. Development of an optimized freeze-drying protocol for OM-PBAE nucleic acid polyplexes. Int J Pharm. 2019;569: Article 118612. 31415876 10.1016/j.ijpharm.2019.118612

[88] Cardle II, Cheng EL, Jensen MC, Pun SH. Biomaterials in chimeric antigen receptor T-cell process development. Acc Chem Res. 2020;53(9):1724–1738. 32786336 10.1021/acs.accounts.0c00335

[89] Smith TT, Stephan SB, Moffett HF, McKnight LE, Ji W, Reiman D, Bonagofski E, Wohlfahrt ME, Pillai SPS, Stephan MT. In situ programming of leukaemia-specific T cells using synthetic DNA nanocarriers. Nat Nanotechnol. 2017;12:813–820. 28416815 10.1038/nnano.2017.57 PMC5646367

[90] Parayath NN, Stephan SB, Koehne AL, Nelson PS, Stephan MT. In vitro-transcribed antigen receptor mRNA nanocarriers for transient expression in circulating T cells in vivo. Nat Commun. 2020;11(1):6080. 33247092 10.1038/s41467-020-19486-2 PMC7695830

[91] Aghajanian H, Kimura T, Rurik JG, Hancock AS, Leibowitz MS, Li L, Scholler J, Monslow J, Lo A, Han W, et al. Targeting cardiac fibrosis with engineered T cells. Nature. 2019;573(7774): 430–433. 31511695 10.1038/s41586-019-1546-z PMC6752964

[92] Zong Y, Lin Y, Wei T, Cheng Q. Lipid nanoparticle (LNP) enables mRNA delivery for cancer therapy. Adv Mater. 2023;35(51):e2303261. 37196221 10.1002/adma.202303261

[93] Cullis PR, Hope MJ. Lipid nanoparticle systems for enabling gene therapies. Mol Ther: J American Soc Gene Ther. 2017;25(7):1467–1475. 10.1016/j.ymthe.2017.03.013 PMC5498813 28412170

[94] Billingsley MM, Hamilton AG, Mai D, Patel SK, Swingle KL, Sheppard NC, June CH, Mitchell MJ. Orthogonal design of experiments for optimization of lipid nanoparticles for mRNA engineering of CAR T cells. Nano Lett. 2022;22(1):533–542. 34669421 10.1021/acs.nanolett.1c02503 PMC9335860

[95] Rurik JG, Tombácz I, Yadegari A, Méndez Fernández PO, Shewale SV, Li L, Kimura T, Soliman OY, Papp TE, Tam YK, et al. CAR T cells produced in vivo to treat cardiac injury. Science. 2022;375(6576):91–96. 34990237 10.1126/science.abm0594 PMC9983611

[96] Udhayakumar VK, De Beuckelaer A, McCaffrey J, McCrudden CM, Kirschman JL, Vanover D, Van Hoecke L, Roose K, Deswarte K, De Geest BG, et al. Arginine-rich peptide-based mRNA Nanocomplexes efficiently instigate cytotoxic T cell immunity dependent on the amphipathic Organization of the Peptide. Adv Healthc Mater. 2017;6(13): Article 1601412. 10.1002/adhm.201601412 28436620

[97] Delivering the Messenger: Advances in Technologies for Therapeutic mRNA Delivery - PubMed, (n.d.). [accessed 18 December 2023] https://pubmed.ncbi.nlm.nih.gov/30846391/10.1016/j.ymthe.2019.02.012 PMC6453548 30846391

[98] Raes L, De Smedt SC, Raemdonck K, Braeckmans K. Non-viral transfection technologies for next-generation therapeutic T cell engineering. Biotechnol Adv. 2021;49: Article 107760. 33932532 10.1016/j.biotechadv.2021.107760

[99] Engineering Efficient CAR-T Cells via Electroactive Nanoinjection - PubMed, (n.d.). [accessed 18 December 2023] https://pubmed.ncbi.nlm.nih.gov/37434421/10.1002/adma.202304122 37434421

[100] Intracellular delivery of messenger RNA by recombinant PP7 virus-like particles carrying low molecular weight protamine - PubMed, (n.d.). [accessed 18 December 2023] https://pubmed.ncbi.nlm.nih.gov/27233770/10.1186/s12896-016-0274-9 PMC4884372 27233770

[101] Chen Y, Pal S, Hu Q. Recent advances in biomaterial-assisted cell therapy. J Mater Chem B. 2022;10:7222–7238. 35612089 10.1039/d2tb00583b

[102] Shen J, Yang D, Ding Y. Advances in promoting the efficacy of chimeric antigen receptor T cells in the treatment of hepatocellular carcinoma. Cancers. 2022;14:5018. 36291802 10.3390/cancers14205018 PMC9599749

[103] Xia Y, Fu S, Ma Q, Liu Y, Zhang N. Application of nano-delivery systems in lymph nodes for tumor immunotherapy. Nano-Micro Lett. 2023;15:145. 10.1007/s40820-023-01125-2 PMC10239433 37269391

[104] Liu L, Ye Q, Wu Y, Hsieh W-Y, Chen C-L, Shen H-H, Wang S-J, Zhang H, Hitchens TK, Ho C. Tracking T-cells in vivo with a new nano-sized MRI contrast agent. Nanomedicine: Nanotechnol Biol Med. 2012;8(8):1345–1354. 10.1016/j.nano.2012.02.017 PMC3383940 22406186

[105] Harmsen S, Medine EI, Moroz M, Nurili F, Lobo J, Dong Y, Turkekul M, Pillarsetty NVK, Ting R, Ponomarev V, et al. A dual-modal PET/near infrared fluorescent nanotag for long-term immune cell tracking. Biomaterials. 2021;269: Article 120630. 33395580 10.1016/j.biomaterials.2020.120630 PMC7870530

[106] Betzer O, Gao Y, Shamul A, Motiei M, Sadan T, Yehuda R, Atkins A, Cohen CJ, Shen M, Shi X, et al. Multifunctional nanoprobe for real-time in vivo monitoring of T cell activation. Nanomedicine. 2022;46: Article 102596. 36031044 10.1016/j.nano.2022.102596

[107] Kim GB, Aragon-Sanabria V, Randolph L, Jiang H, Reynolds JA, Webb BS, Madhankumar A, Lian X, Connor JR, Yang J, et al. High-affinity mutant Interleukin-13 targeted CAR T cells enhance delivery of clickable biodegradable fluorescent nanoparticles to glioblastoma. Bioact Mater. 2020;5(3):624–635. 32405577 10.1016/j.bioactmat.2020.04.011 PMC7212185

[108] Duwa R, Pokhrel RH, Banstola A, Pandit M, Shrestha P, Jeong J-H, Chang J-H, Yook S. T-cell engaging poly(lactic-co-glycolic acid) nanoparticles as a modular platform to induce a potent cytotoxic immunogenic response against PD-L1 overexpressing cancer. Biomaterials. 2022;291: Article 121911. 36399833 10.1016/j.biomaterials.2022.121911

[109] Li X, Lovell JF, Yoon J, Chen X. Clinical development and potential of photothermal and photodynamic therapies for cancer. Nat Rev Clin Oncol. 2020;17(11):657–674. 32699309 10.1038/s41571-020-0410-2

[110] Jung HS, Verwilst P, Sharma A, Shin J, Sessler JL, Kim JS. Organic molecule-based photothermal agents: An expanding photothermal therapy universe. Chem Soc Rev. 2018;47(7):2280–2297. 29528360 10.1039/c7cs00522a PMC5882556

[111] Chen Z, Pan H, Luo Y, Yin T, Zhang B, Liao J, Wang M, Tang X, Huang G, Deng G, et al. Nanoengineered CAR-T biohybrids for solid tumor immunotherapy with microenvironment photothermal-remodeling strategy. Small. 2021;17(14):2007494. 10.1002/smll.202007494 33711191

[112] Pardhi E, Yadav R, Chaurasiya A, Madan J, Guru SK, Singh SB, Mehra NK. Multifunctional targetable liposomal drug delivery system in the management of leukemia: Potential, opportunities, and emerging strategies. Life Sci. 2023;325: Article 121771. 37182551 10.1016/j.lfs.2023.121771

[113] Hua S, Wu SY. The use of lipid-based nanocarriers for targeted pain therapies. Front Pharmacol. 2013;4:143. 24319430 10.3389/fphar.2013.00143 PMC3836271

[114] Yang M, Li J, Gu P, Fan X. The application of nanoparticles in cancer immunotherapy: Targeting tumor microenvironment. Bioact Mater. 2021;6:1973–1987. 33426371 10.1016/j.bioactmat.2020.12.010 PMC7773537

[115] Fernandez-Fernandez A, Manchanda R, Kumari M. Lipid-engineered nanotherapeutics for cancer management. Front Pharmacol. 2023;14:1125093. 37033603 10.3389/fphar.2023.1125093 PMC10076603

[116] Taléns-Visconti R, Díez-Sales O, de Julián-Ortiz JV, Nácher A. Nanoliposomes in cancer therapy: Marketed products and current clinical trials. Int J Mol Sci. 2022;23(8):4249. 35457065 10.3390/ijms23084249 PMC9030431

[117] Hatakeyama H, Akita H, Harashima H. The polyethyleneglycol dilemma: Advantage and disadvantage of PEGylation of liposomes for systemic genes and nucleic acids delivery to tumors. Biol Pharm Bull. 2013;36(6):892–899. 23727912 10.1248/bpb.b13-00059

[118] Yingchoncharoen P, Kalinowski DS, Richardson DR. Lipid-based drug delivery systems in cancer therapy: What is available and what is yet to come. Pharmacol Rev. 2016;68(3):701–787. 27363439 10.1124/pr.115.012070 PMC4931871

[119] Zhang F, Stephan SB, Ene CI, Smith TT, Holland EC, Stephan MT. Nanoparticles that reshape the tumor milieu create a therapeutic window for effective T-cell therapy in solid malignancies. Cancer Res. 2018;78(13):3718–3730. 29760047 10.1158/0008-5472.CAN-18-0306 PMC6030470

[120] Huang L, Li Y, Du Y, Zhang Y, Wang X, Ding Y, Yang X, Meng F, Tu J, Luo L, et al. Mild photothermal therapy potentiates anti-PD-L1 treatment for immunologically cold tumors via an all-in-one and all-in-control strategy. Nat Commun. 2019;10(1):4871. 31653838 10.1038/s41467-019-12771-9 PMC6814770

[121] Rodríguez F, Caruana P, De La Fuente N, Español P, Gámez M, Balart J, Llurba E, Rovira R, Ruiz R, Martín-Lorente C, et al. Nano-based approved pharmaceuticals for cancer treatment: Present and future challenges. Biomol Ther. 2022;12(6):784. 10.3390/biom12060784 PMC9221343 35740909

[122] Obozina AS, Komedchikova EN, Kolesnikova OA, Iureva AM, Kovalenko VL, Zavalko FA, Rozhnikova TV, Tereshina ED, Mochalova EN, Shipunova VO. Genetically encoded self-assembling protein nanoparticles for the targeted delivery in vitro and in vivo. Pharmaceutics. 2023;15(1):231. 36678860 10.3390/pharmaceutics15010231 PMC9861179

[123] Chen Z, Liu L, Liang R, Luo Z, He H, Wu Z, Tian H, Zheng M, Ma Y, Cai L. Bioinspired hybrid protein oxygen nanocarrier amplified photodynamic therapy for eliciting anti-tumor immunity and Abscopal effect. ACS Nano. 2018;12(8):8633–8645. 30005164 10.1021/acsnano.8b04371

[124] Xu J, Ma Q, Zhang Y, Fei Z, Sun Y, Fan Q, Liu B, Bai J, Yu Y, Chu J, et al. Yeast-derived nanoparticles remodel the immunosuppressive microenvironment in tumor and tumor-draining lymph nodes to suppress tumor growth. Nat Commun. 2022;13(1):110. 35013252 10.1038/s41467-021-27750-2 PMC8748771

[125] Tang L, Zheng Y, Melo MB, Mabardi L, Castaño AP, Xie Y-Q, Li N, Kudchodkar SB, Wong HC, Jeng EK, et al. Enhancing T cell therapy through TCR-signaling-responsive nanoparticle drug delivery. Nat Biotechnol. 2018;36(8): 707–716. 29985479 10.1038/nbt.4181 PMC6078803

[126] Trofimov AD, Ivanova AA, Zyuzin MV, Timin AS. Porous inorganic carriers based on silica, calcium carbonate and calcium phosphate for controlled/modulated drug delivery: Fresh outlook and future perspectives. Pharmaceutics. 2018;10:167. 30257514 10.3390/pharmaceutics10040167 PMC6321143

[127] Biodegradable Inorganic Nanostructured Biomaterials for Drug Delivery - Cai - 2020 - Advanced Materials Interfaces - Wiley Online Library, (n.d.). [accessed 18 December 2023] https://onlinelibrary.wiley.com/doi/10.1002/admi.202000819

[128] Trushina DB, Borodina TN, Belyakov S, Antipina MN. Calcium carbonate vaterite particles for drug delivery: Advances and challenges. Mater Today Adv. 2022;14: Article 100214. 36785703 10.1016/j.mtadv.2022.100214 PMC9909585

[129] Maleki Dizaj S, Sharifi S, Ahmadian E, Eftekhari A, Adibkia K, Lotfipour F. An update on calcium carbonate nanoparticles as cancer drug/gene delivery system. Expert Opin Drug Deliv. 2019;16(4):331–345. 30807242 10.1080/17425247.2019.1587408

[130] Liu J, Cui L, Losic D. Graphene and graphene oxide as new nanocarriers for drug delivery applications. Acta Biomater. 2013;9(12):9243–9257. 23958782 10.1016/j.actbio.2013.08.016

[131] Itoo AM, Vemula SL, Gupta MT, Giram MV, Kumar SA, Ghosh B, Biswas S. Multifunctional graphene oxide nanoparticles for drug delivery in cancer. J Control Release. 2022;350:26–59. 35964787 10.1016/j.jconrel.2022.08.011

[132] Abánades Lázaro I, Forgan RS. Application of zirconium MOFs in drug delivery and biomedicine. Coord Chem Rev. 2019;380:230–259.

[133] Zhu H, Zheng J, Oh XY, Chan CY, Low BQL, Tor JQ, Jiang W, Ye E, Loh XJ, Li Z. Nanoarchitecture-integrated hydrogel systems toward therapeutic applications. ACS Nano. 2023;17(9):7953–7978. 37071059 10.1021/acsnano.2c12448

[134] Sepantafar M, Maheronnaghsh R, Mohammadi H, Radmanesh F, Hasani-sadrabadi MM, Ebrahimi M, Baharvand H. Engineered hydrogels in cancer therapy and diagnosis. Trends Biotechnol. 2017;35(11):1074–1087. 28734545 10.1016/j.tibtech.2017.06.015

[135] Li X, Xu X, Xu M, Geng Z, Ji P, Liu Y. Hydrogel systems for targeted cancer therapy. Front Bioeng Biotechnol. 2023;11:1140436. 36873346 10.3389/fbioe.2023.1140436 PMC9977812

[136] Da Silva LP, Cerqueira MT, Sousa RA, Reis RL, Correlo VM, Marques AP. Engineering cell-adhesive gellan gum spongy-like hydrogels for regenerative medicine purposes. Acta Biomater. 2014;10(11):4787–4797. 25048775 10.1016/j.actbio.2014.07.009

[137] Jeon EY, Choi D, Choi S, Won J, Jo Y, Kim H, Jung Y, Shin SC, Min H, Choi HW, et al. Enhancing adoptive T-cell therapy with fucoidan-based IL-2 delivery microcapsules. Bioeng Transl Med. 2023;8(1): Article e10362. 36684086 10.1002/btm2.10362 PMC9842027

[138] Hu Q, Li H, Archibong E, Chen Q, Ruan H, Ahn S, Dukhovlinova E, Kang Y, Wen D, Dotti G, et al. Inhibition of post-surgery tumour recurrence via a hydrogel releasing CAR-T cells and anti-PDL1-conjugated platelets. Nat Biomed Eng. 2021;5(9):1038–1047. 33903744 10.1038/s41551-021-00712-1 PMC9102991

[139] Tan J, Luo Y, Guo Y, Zhou Y, Liao X, Li D, Lai X, Liu Y. Development of alginate-based hydrogels: Crosslinking strategies and biomedical applications. Int J Biol Macromol. 2023;239: Article 124275. 37011751 10.1016/j.ijbiomac.2023.124275

[140] Reig-Vano B, Tylkowski B, Montané X, Giamberini M. Alginate-based hydrogels for cancer therapy and research. Int J Biol Macromol. 2021;170:424–436. 33383080 10.1016/j.ijbiomac.2020.12.161

[141] Chao Y, Wei T, Li Q, Liu B, Hao Y, Chen M, Wu Y, Song F, Chen Q, Liu Z. Metformin-containing hydrogel scaffold to augment CAR-T therapy against post-surgical solid tumors. Biomaterials. 2023;295: Article 122052. 36827893 10.1016/j.biomaterials.2023.122052

[142] Bhatta R, Han J, Liu Y, Bo Y, Wang H. T cell-responsive macroporous hydrogels for in situ T cell expansion and enhanced antitumor efficacy. Biomaterials. 2023;293: Article 121972. 36566554 10.1016/j.biomaterials.2022.121972 PMC9868092

[143] Mao Y, Liu J, Shi T, Chen G, Wang S. A novel self-assembly nanocrystal as lymph node-targeting delivery system: Higher activity of lymph node targeting and longer efficacy against lymphatic metastasis. AAPS PharmSciTech. 2019;20(7):292. 31428888 10.1208/s12249-019-1447-3

[144] Lin J, Hsu C, Chen J, Fang Z, Chen H, Yao B, Shiau GHM, Tsai J, Gu M, Jung M, et al. Facile transformation of murine and human primary dendritic cells into robust and modular artificial antigen-presenting systems by intracellular hydrogelation. Adv Mater. 2021;33(30):2101190. 10.1002/adma.202101190 34096117

[145] Hu Y, Niemeyer CM. From DNA nanotechnology to material systems engineering. Adv Mater. 2019;31(26):1806294. 10.1002/adma.201806294 30767279

[146] Douglas SM, Dietz H, Liedl T, Högberg B, Graf F, Shih WM. Self-assembly of DNA into nanoscale three-dimensional shapes. Nature. 2009;459:414–418. 19458720 10.1038/nature08016 PMC2688462

[147] Madsen M, Gothelf KV. Chemistries for DNA nanotechnology. Chem Rev. 2019;119(10):6384–6458. 30714731 10.1021/acs.chemrev.8b00570

[148] Huang X, Williams JZ, Chang R, Li Z, Burnett CE, Hernandez-Lopez R, Setiady I, Gai E, Patterson DM, Yu W, et al. DNA scaffolds enable efficient and tunable functionalization of biomaterials for immune cell modulation. Nat Nanotechnol. 2021;16(2):214–223. 33318641 10.1038/s41565-020-00813-z PMC7878327

[149] Lyu S, Dong Z, Xu X, Bei H-P, Yuen H-Y, James Cheung C-W, Wong M-S, He Y, Zhao X. Going below and beyond the surface: Microneedle structure, materials, drugs, fabrication, and applications for wound healing and tissue regeneration. Bioact Mater. 2023;27:303–326. 37122902 10.1016/j.bioactmat.2023.04.003 PMC10140753

[150] Moreira AF, Rodrigues CF, Jacinto TA, Miguel SP, Costa EC, Correia IJ. Microneedle-based delivery devices for cancer therapy: A review. Pharmacol Res. 2019;148: Article 104438. 31476370 10.1016/j.phrs.2019.104438

[151] Li H, Wang Z, Ogunnaike EA, Wu Q, Chen G, Hu Q, Ci T, Chen Z, Wang J, Wen D, et al. Scattered seeding of CAR T cells in solid tumors augments anticancer efficacy. Natl Sci Rev. 2022;9(3):nwab172. 35265340 10.1093/nsr/nwab172 PMC8900686

[152] Shayan M, Chun Y. An overview of thin film nitinol endovascular devices. Acta Biomater. 2015;21:20–34. 25839120 10.1016/j.actbio.2015.03.025

[153] Coon ME, Stephan SB, Gupta V, Kealey CP, Stephan MT. Nitinol thin films functionalized with CAR-T cells for the treatment of solid tumours. Nat Biomed Eng. 2019;4:195–206. 31819155 10.1038/s41551-019-0486-0

[154] Du H, Bartleson JM, Butenko S, Alonso V, Liu WF, Winer DA, Butte MJ. Tuning immunity through tissue mechanotransduction. Nat Rev Immunol. 2023;23:174–188. 35974148 10.1038/s41577-022-00761-w PMC9379893

[155] Huse M. Mechanical forces in the immune system. Nat Rev Immunol. 2017;17(11):679–690. 28757604 10.1038/nri.2017.74 PMC6312705

[156] Xi W, Saw TB, Delacour D, Lim CT, Ladoux B. Material approaches to active tissue mechanics. Nat Rev Mater. 2019;4:23–44.

[157] Li X, Shou Y, Tay A. Hydrogels for engineering the immune system. Adv NanoBiomed Res. 2021;1(3):2000073.

[158] Ma L, Dichwalkar T, Chang JYH, Cossette B, Garafola D, Zhang AQ, Fichter M, Wang C, Liang S, Silva M, et al. Enhanced CAR–T cell activity against solid tumors by vaccine boosting through the chimeric receptor. Science. 2019;365(6449):162–168. 31296767 10.1126/science.aav8692 PMC6800571

[159] Tang X, Yang Y, Zheng M, Yin T, Huang G, Lai Z, Zhang B, Chen Z, Xu T, Ma T, et al. Magnetic–acoustic sequentially actuated CAR T cell microrobots for precision navigation and in situ antitumor immunoactivation. Adv Mater. 2023;35(18):2211509. 10.1002/adma.202211509 36807373

[160] Agarwalla P, Ogunnaike EA, Ahn S, Froehlich KA, Jansson A, Ligler FS, Dotti G, Brudno Y. Bioinstructive implantable scaffolds for rapid in vivo manufacture and release of CAR-T cells. Nat Biotechnol. 2022;40(8):1250–1258. 35332339 10.1038/s41587-022-01245-x PMC9376243

[161] Adu-Berchie K, Mooney DJ. Biomaterials as local niches for immunomodulation. Acc Chem Res. 2020;53(9):1749–1760. 32786230 10.1021/acs.accounts.0c00341

[162] Zhu L, Liu J, Zhou G, Liu T, Dai Y, Nie G, Zhao Q. Remodeling of tumor microenvironment by tumor-targeting nanozymes enhances immune activation of CAR T cells for combination therapy. Small. 2021;17(43):2102624. 10.1002/smll.202102624 34378338

